# Unveiling Emergence and Holism in Biology: Essential Insights from Self-Organization

**DOI:** 10.3390/biology15070579

**Published:** 2026-04-04

**Authors:** Srdjan Kesić

**Affiliations:** Department of Neurophysiology, Institute for Biological Research “Siniša Stanković”—National Institute of the Republic of Serbia, University of Belgrade, Despot Stefan Blvd., 142, 11108 Belgrade, Serbia; srdjan.kesic@ibiss.bg.ac.rs; Tel.: +381-11-2078-300

**Keywords:** self-organization, emergence, holism, theories, biology

## Abstract

This essay re-examines the enduring conceptual landscape of *self-organization* with the objective of clarifying the fundamental role of *emergence* and *holism* in contemporary biology. A deeper appreciation of these concepts can help biologists establish a solid theoretical foundation, avoiding biases that may occur when such perspectives are incomplete. The essay begins by exploring the historical roots of *self-organization* in biology and then discusses relevant theories of *self-organization* in the biological sciences. A comprehensive understanding of these historical foundations and the significance of *self-organization* is used to explore and conceptually connect *self-organization* to *emergence* and *holism*. Throughout the essay, these theoretical frameworks are illustrated with significant examples from biology and other scientific fields. In conclusion, the essay emphasizes the need for deeper conceptual reflection to clarify many controversial issues at the intersection of *self-organization*, *emergence*, and *holism* in biology and, more broadly, within science.

## Simple Summary

This study offers unique insights into self-organization, emergence, and holism, supported by examples from biology. It examines these conceptual frameworks to clarify them and make them more relevant to biologists, scientists, and philosophers. The article addresses these issues from both historical and current perspectives and concludes that further insights are needed to advance theoretical and philosophical understanding of systems and holistic biology.

## 1. Introduction

I will begin with an unconventional introduction. What is the purpose of this lengthy paper? Before addressing that question, let me pose another: Can biology exist without its foundational concepts, without fundamental theories such as *thermodynamics*, Prigogine’s idea of dissipative structures, and Varela and Maturana’s theory of *autopoiesis*? Furthermore, can we exclude crucial concepts like *self-organization*, *emergence*, and *holism* from contemporary systems biology? If the answer is no, then this paper makes sense. It is intended as a valuable conceptual guide for working scientists, undergraduate and graduate students, and biologists deeply involved in laboratory work, and those interdisciplinary-oriented scientists and philosophers managing their careers while meeting project demands. They often lack the time to thoroughly explore databases, books, and journals to understand these conceptual issues or apply them to their daily research.

With this in mind, this paper aims to critically engage with these conceptual and theoretical foundations while presenting examples from the biological sciences that vividly illustrate them. Rather than simply asserting the significance of philosophy in shaping biologists’ *theoretical perspectives*, I believe that presenting and exploring these theories and concepts—both in historical and contemporary contexts—will provide more practical benefits. Biologists and others will have the opportunity to discover essential insights within this paper and the referenced works, ultimately enriching their understanding and enhancing their research.

Concepts such as ‘holism’, ‘order’, ‘synergy’, and ‘chaos’ were discussed in Ancient Greek philosophy and throughout the history of philosophy before being adopted for scientific use in modern theories of *self-organization* and complexity [1,2,3,4]. Few scientific or philosophical terms today generate as much discussion as “system” and “complexity”, along with other concepts such as “nonlinearity,“ self-organization, and “emergence”. These scientific ideas, with deep philosophical roots, highlight the significant role that philosophy plays in shaping the theory and practice of complex systems science. As Woermann et al. [5] (p. 1) state, “A rigorous understanding of the nature and implications of complexity reveals that the underlying assumptions informing our understanding of complex phenomena are deeply related to general philosophical issues.” The eclectic and speculative nature of the “philosophy of complexity” is a fundamental aspect of complexity research [6]. There is little doubt about this.

Considering a strong reliance of today’s biology on complexity science, advancing biological knowledge in the era of artificial intelligence is meaningful only if the conceptual foundations of complexity are clearly specified and made explicit [7,8,9,10]. Philosophy, as a strong “protoscience generator”, can facilitate this task by promoting interdisciplinary and multidisciplinary contact between complexity scientists and biologists [11,12].

I have also noticed a lack of literature explicitly linking *self-organization* in biology to its historical roots. Additionally, philosophical papers on *emergence* and *holism* are often highly speculative, complex, and difficult for biologists to understand. This observation has motivated me to structure this paper accordingly, incorporating examples from microbiology, genetics, molecular biology, cell biology, and physiology that are more familiar to average biologists. I believe that, given the paper’s structure and the volume of information presented, it may also serve as a reference point for philosophers. Thus, the main thread of this paper is to further illustrate the connections among the concepts of *self-organization*, *emergence*, and *holism*.

This article is organized as follows. First, in Section 2, I raise concerns about the meaning of the term “complex biological system”. Section 3 introduces the five historical-theoretical roots relevant to thinking about self-organized biological systems. In Section 4, I discuss some important, though not all, theories of *self-organization*, some of which originate in biology and have found their place across science and practical applications. Section 5 introduces the basics of *emergence*, shows its relevance to emergent biological systems, and then examines the significant implications of *self-organization* and *emergence* for a holistic understanding of organisms and holistic biology. These sections allow us to draw some conclusions and suggest directions for further inquiry.

## 2. What Is a Complex Biological System?

To begin this paper, it is important to become familiar with *self-organization*, *emergence*, and *holism* by first exploring the concept of complex systems and the nature of complexity. This foundation will support further discussion of a complexity-oriented approach to biology that emphasizes self-organized pattern formation. Mazzocchi [13], p. 1 identifies at least two types of complex systems: “(i) one mainly characterized by the generation of stable patterns through self-reinforcing dynamics at the lower levels (Bénard convection), and (ii) a distinct type characterized by a more complex organization that makes them ‘minimally decomposable’ and demonstrates autonomy (living systems)”. *Rayleigh–Bénard convection* (RB) in fluid dynamics describes the behavior of a fluid between two thermally conducting plates. The fluid is heated from below, creating a temperature difference [14]. Initially, this movement is disorganized and uncoordinated, allowing energy transfer between the lower and upper plates. However, once a certain temperature gradient is reached, the movement becomes more organized and coordinated [14]. The RB is of great significance for a better understanding of many processes in physics, meteorology, physical and industrial chemistry, and engineering, helping solve practical problems.

For example, Pelusi et al. [15,16] provide a comprehensive numerical simulation of dynamic emulsion behavior in conditions of convective flows. This type of experimentation has been proven essential for understanding the behavior of emulsions (soft materials) across various spatial and temporal scales. Their findings show an interaction between the structural and rheological complexity of emulsions and the dynamics influenced by convective flows.

The second type of system, relating to “autonomic” living systems, was formalized by Maturana and Varela’s *autopoiesis* theory [17,18,19,20], and has now been reconceptualized as the process-based theory of autonomy (see [21]). The main idea is that a living system can self-organize and self-determine, maintaining its structure despite a challenging external environment [21,22,23]. Nevertheless, this useful division still does not fully answer the question of what precisely distinguishes a complex system, or the science that studies it, from a simple system.

There is no consensus on a concise definition of a complex system, on the term “system”, or on the disciplinary status of the science of complexity (sciences of interconnectedness) [24,25,26,27,28]. This, among other things, can make it hard to clearly differentiate science from pseudoscience in complexity studies, lowering the credibility of that field with mainstream scientists [24]. However, one should not be alarmed by these unresolved dilemmas. First, the science of complexity is advancing faster than philosophical, meta-scientific, and cognitive frameworks can reflect it or keep pace with it [29]. Indeed, the complexity paradigm has significantly reshaped science, engineering, and society [30,31,32,33,34,35,36]. It has prompted new questions, the exploration of new possibilities, and the development of innovative solutions in a world of constant change [37,38,39]. Therefore, even if it is just the “paradigm,” it is a good one, transforming the sciences, such as biology, and moving our focus from “analytic” to more “synthetic-holistic views” of complexity [40].

Second, the application of complex systems science to chemical and biological systems is relatively new and in its early stages compared to traditional reductionist approaches that insist on knowledge of the base (e.g., molecular machinery of the cell) [41]. Chaos and complexity theory profoundly alter our worldview by challenging the long-standing reductionist and mechanistic scientific model established by Galileo, Descartes, and Newton in the 16th and 17th centuries, which influenced much of science until the turn of this century. This framework, to which many scientists anchor their theoretical perspectives and which upholds the unquestionable value of universal scientific laws, is increasingly regarded as inadequate for explaining the intricacies of complex reality, including *biocomplexity* [42,43,44]. Third, the vagueness in the definition of what is a complex system and what makes complexity science distinguishable field of research can be described through the term “generous Darwinian fog”, which is recently used in microbiology to describe a situation where we should not make a “concept too tightly with a rigid definition at an early stage of its development” [45], p. 8.

Despite the lack of consensus on defining complex systems, there is some agreement on their common characteristics. In cybernetics, complex systems are those whose components are organized and interconnected through feedback loops [46] and directed by “control information” [47]. These interconnected components interact nonlinearly, making it difficult to predict system behaviour from knowledge of the components alone. As proposed by chaos theory, *nonlinearity* means that a system’s output is not directly proportional to its input [48,49]. In other words, it is a key feature of *systemic dynamism* that underpins contextual sensitivity to initial conditions [50], pp. 80–81. Various combinations of nonlinear interactions can create numerous feedback loops, resulting in new system states or emergent properties at multiple scales [51]. Holland [52] and Barnett [28], for example, argue that although both complex and complicated systems consist of many interconnected components, only complex systems exhibit *emergence*.

Many theories of complexity, including *General Systems Theory*, *Complex Adaptive Systems*, and *Synergetics*, recognize the holistic principle that the “whole is greater than the sum of its parts.” In addition to feedback loops, *nonlinearity*, and *emergence*, *holism* stands out as the fifth crucial philosophical framework for understanding complex systems, including biological ones. With advancements in computational and information sciences, this list can also be expanded to encompass modeling, simulation, and the computability of system properties. In a more computational context, Timothy Allen [53], p. 39 argues that a system is complex if not all its constituent models are simulable, which makes distinguishing between simple and complex systems an all-or-nothing proposition. Expanding our list is not yet complete. Recent insights from complexity studies begin to consider the causal role of constraints in complex systems [54]. Perhaps other characteristics are discussed by certain groups or intellectual circles regarding complexity in natural and socio-technical systems. However, we want to get into them and will focus on some of them listed above.

Where is self-organization on this list? Self-organization is an umbrella term for operationalizing these principles in the context of system development and evolution within a defined space and time. This operationalization within any given system rests on Mario Bunge’s “ontological systematism”, the belief that everything is formed by systems of systems, i.e., the stance that reality is composed of subsystems inspired by Paul Henri Thiry d’Holbach’s *Système de la Nature* (published in 1770) and Ludwig von Bertalanffy’s *General System Theory* [55,56]. These subsystems possess relative autonomy in their development and dynamics, yet they are interrelated and interconnected [56]. As physics and computation show, *self-organization* is driven by quantum mechanics and physico-chemical principles, and constrained by environmental “top-down” influences that produce and maintain these subsystems, accounting for their interrelatedness and adaptability.

Let us examine how these concepts and principles are effectively applied in the field of systems complexity science, first broadly and then more specifically, by step-by-step exposure in the continuation of the paper. The rule, or “dynamic,” which specifies how the system evolves, and the initial condition, or “state,” from which the system begins to evolve, are two critical components of the often-used syntagma “dynamic system” [57]. These systems are also characterized by the existence of one or more ‘attractors’ that serve as focal points for understanding system behaviour [50]. Attractors become “strange” if behaviour is continually drawn towards them, but never through precisely the same pathways, and never to the extent that a long-term equilibrium state is reached [50], pp. 80–81. These “strange” behavioural trajectories can result in fundamentally unpredictable behaviour, sensitized to a specific array of contextual features and issues, which, according to Cooksey [50], p. 81, Loye [58], and Briggs and Peat [59], allows the creative harnessing of positive system feedback to achieve a new, more stable behavioural pattern.

Complex systems that have undergone chaotic phases are referred to as “self-organizing” or “emergent” systems [50]. These systems are predictable in the short term because it is possible to predict values of time series in a limited way—but not in the long term (see [50,60]). These chaotic phases are accompanied by multiple positive and negative feedback loops. Schueler [60], p. 3 refers to them as *feedback mechanisms* that provide the “means by which matter, energy, or information are fed back into the system”. This space-time-dependent chaotic behavior may lead to instability, which is further amplified by positive feedback mechanisms, ultimately shifting the system towards a new, self-organized, time-limited state of stability. This series of events clearly shows that the system has all the necessary elements to evolve and continually adapt its composition in an unpredictable environment.

Wu et al. [61], p. 9 argue that contemporary theories of complex self-organizing systems exemplify effective theory because they reveal the inherent unity of time and space. These theories, together with the continually expanding mathematical frameworks developed for them and based on them, unify the complex relationships involved in spatiotemporal transformation by emphasizing the “spatialization of time” and the “temporalization of space” in the evolution of interactions. This dynamic, self-organizing view of nature introduces a non-static, energetic, and relational approach to complexity-oriented biology.

A complex system is not simply a mind-independent object or entity waiting to be easily abstracted, discovered, and described. Our own minds—understood through von Foerster’s second-order cybernetic dictum ‘role of the observer’—must be actively engaged in formulating and understanding it, even though complex interactions may seriously obscure reality [43,62,63]. In other words, epistemological, methodological, and technical hurdles stand in the way of any attempt to portray and study complexity and to give it meaningful context. The exact meaning of complexity should be sought in the meaning a subjective observer extracts from the pattern or sequence [64,65,66]. To try to keep pace with these challenges, Mikulecky [42], p. 341 explicitly called on the scientific community to reconsider dismantling methodological, epistemological, and cognitive barriers to address complexity, stating that “complexity science demands that the barriers and constraints be removed to gain a more complete view of nature.” More about the boundaries of a complex, holistic system will be discussed in the section on holism.

In biology, these theories of *self-organization* and complexity require a high level of intellectual rigour and a cognitive shift from simplicity to complexity [61]. This, together with other changes in our mindset and scientific practice, shifts the focus from the role of selection in the evolution of organisms to the *nonequilibrium thermodynamics* underlying the emergence of life on Earth. In this revitalized and updated scientific context, which insists on the physics of emergence, the central question becomes how to explain the:

“Meshing together of upward and downward causation that aligns with the underlying physics” to provide a comprehensive search for and explanation of the origin and evolution of life [67], p. 1.

In this context, revitalized means that physics and mathematics have long been associated with biology. However, in the field of biocomplexity, these disciplines have undergone computational transformations that are now increasingly accessible to biologists. Thus, this new physics and mathematics seem to offer biology far more than they did 50 or more years ago. Kauffman [68] summarized this conceptual, cognitive, and ideological transition in one sentence:

“The spontaneous order in complex systems implies that selection may not be the sole source of order in organisms, and that we must invent a new theory of evolution which encompasses the marriage of selection and self-organization.”

Alexander Rosenberg [69], together with others, responds to Mikulecký’s and similar appeals to dismantle the cognitive and technical barriers when faced with complexity by urging us to trust in the far-reaching power of the laws of physics. Lineweaver et al. [70] express these beliefs and expectations: “beneath the surface complexity of the universe lies an elegant mathematical simplicity.” However, Rosenberg adopts a temporarily sceptical stance, arguing that our current cognitive and computational capacities are too limited to address biocomplexity effectively [69]. This is the main reason the field operates pragmatically, far from the precision of fundamental physics.

By taking cognitive limitations seriously, Brian Johnson [29] argues that, because our brain cannot think in parallel, we cannot intuitively grasp the emergence arising from the many nonlinear interactions occurring simultaneously in time and space. This raises the credibility of those who claim that emergence is nothing more than a ‘mysterious’ and confusing concept that limits scientific progress and our understanding of the behaviour of cellular automata and of natural and social pattern formation. Perhaps, by finding ways to improve cognitive abilities with help from parallel-processing computers and, nowadays, artificial intelligence, we can better understand emergent phenomena, concludes Johnson. This explains why *computational emergence*, the acquisition of properties through computation, needs to be taken seriously [71].

These are the genuine aspirations of sincere physicalists and moderate reductionists, not those naive individuals who casually declare that biology is merely a special case of physics as it currently stands. Thoughtful biologists, philosophers, and physicists acknowledge the limitations of current physical laws and the technology required to fully recreate holistic living systems and strive to overcome these challenges. Their moderation enables the study of complex systems and prompts critical questioning of physically supported theories of self-organization in biology.

In this context, the recent, more or less successful, application of mathematics and computation to complex biological systems is gradually changing the characterization of biology as “instrumental” and “mathematically insensitive,” perhaps restoring the conviction that the fundamental laws of physics may be sufficient to explain living hierarchies from molecules to the biosphere. Complexity theorists and biologists go beyond Rosenberg’s “lack of cognitive capacity and means” because most, if not all, agree that “complexity of a system is not necessarily the result of our lack of information” [72], p. 10, and share Rosen’s [73,74] view of complexity as an intrinsic property of a self-organized system. Consequently, life itself is a property of a living system (an organism), not caused by the physical nature of its components, as reductionists often claim, but rather emerges as a “consequence of complex organization of a certain type in a material system” [75], p. 399.

## 3. Self-Organized Complex Biological Systems

In this section, I will first discuss self-organized biological systems. In the next section, I will examine theories of *self-organization*, focusing on their relevance to the biological sciences in distinct ways to provide a basis for conceptually connecting *self-organization* with *emergence* and *holism*. This discussion of the fundamentals of *self-organization* will help readers better understand the biological examples of *emergence* and *holism* presented later in the paper.

The concept of *self-organization* has existed in science and biology, explicitly or implicitly, even before W. Ross Ashby, H. von Foerster, and N. Wiener, among others, began using the term in cybernetics [14]. Haken and Portugali [66], p. 1 make it clear what it means:

“Self-organization is a process by which the interaction between the parts of a complex system gives rise to the spontaneous emergence of patterns, structures, or functions. In this interaction, the system elements exchange matter, energy, and information.”

Wedlich-Söldner and Betz [76] present a similar definition:

“Self-organization refers to the emergence of an overall order in time and space of a given system that results from the collective interactions of its individual components.”

As a curiosity, the explicitly named science of *self-organization*, called “selforganizology,” is perhaps of recent origin (see [77]).

A defining characteristic of all theories of *self-organization* is their commitment to explaining *order* and *organization* from within the system. However, one theory may deviate from this general principle. A longtime friend and collaborator of Herman Haken, Juval Portugali [78], suggests that Haken, in the later stages of *synergetics*, anticipated “top-down” processes in his theory (e.g., the brain operates in a “top-down” manner). This is a valuable resource for current systems biologists who take “top-down” causality seriously, although many philosophers, such as Jaegwon Kim, reject it outright. Any prospective framework that acknowledges internal mechanisms of *self-organization*, modified by external influences, may lead to reconciliation between the *thermodynamics of evolution* and the *Darwinian theory of natural selection* (see [79]). Alternatively, it may help to close the gap between the organism and the environment.

To deepen our understanding of the following concepts, it is important to distinguish between two processes that create macromolecular structures: *self-assembly* and *self-organization*. *Self-assembly* refers to the physical association of molecules into a stable equilibrium structure [80]. For example, in viruses, the assembly of infectious bacteriophage particles includes specific interactions between proteins, nucleic acids, and lipids that result in a steady-state structure [80,81]. It seems that *self-assembly* of cellular biomolecular structures, such as actin, amyloid beta formation in Alzheimer’s disease cell is driven by the increase in the entropy of the system; the release of ordered water (lower entropy) that is abounded in cells to the bulk solvent (higher entropy) is the driving mechanism of assembly [82,83,84]. Molecular assembly depends on many factors, including the functional groups present, the solvent type, the temperature at which the molecules assemble, and the concentration of the building blocks [85]. Even more, neurobiologists consider many other molecular changes in the brain associated with ageing and neurodegenerative diseases, such as Parkinson’s disease and Alzheimer’s, to result from increased entropy [82].

In contrast, *self-organization* pertains to cellular structures such as mitochondria, nuclear subcompartments, the endoplasmic reticulum, and exocytic and endocytic compartments, among others, which are open to the exchange of matter and energy and are governed by steady-state dynamics [80].

Historically, the self-organization in biology has four sources or roots. The first is thermodynamic–physical; the second is physico-chemical oscillatory dynamics; the third is based on the theory of autopoiesis; the fourth relates to the rise of systems biology; and the fifth is based on merging *self-organization* and *information*. The first was articulated in different ways by Ervin Bauer and Ilya Prigogine. The second rests on the discovery of the so-called Belousov–Zhabotinsky (BZ) reaction, while the third builds upon Varela’s and Maturana’s theory of *autopoiesis*. This third root builds upon the first two and, in more recent revisions and expansions—such as the *enactive* and *agential* approach to *organizational biology*—is open to acknowledging “top-down” effects from higher to lower levels and to paying attention to constraints arising from these higher levels [86,87,88]. The fourth is characterized by the rapid development of systems biology. The fifth concerns the *information-computational-theoretic framework* [65,89]. All these roots are interrelated and interwoven, and in today’s systems biology age, they converge.

### 3.1. The First Root: Bauer’s Theoretical Biology and Prigogine’s Dissipative Structures

To understand the first root, let us discuss the groundbreaking and historically significant work of Bauer and Prigogine that ties the fields of *thermodynamics* (physics) and biology together. Most authors usually identify *self-organization* as stemming from cybernetics. However, even before cybernetics, theoretical biologist Ervin Bauer, who was the first to develop a general molecular-based biological theory [90,91], proposed in 1920 [90] and 1935 [91] a highly innovative idea or basic principle of life for that time [92], p. 1; [93].

“Living systems are never in equilibrium; at the expense of their free energy, they constantly perform work to avoid the equilibrium required by the laws of physics and chemistry under existing external conditions.”

What I find interesting is that authors who have examined Bauer’s influential work, such as Elek and Müller [92] and Igamberdiev [94], have reached the same conclusion: Bauer’s ideas are distinct from the *non-equilibrium thermodynamics* of irreversible processes based on the universality of the *Second Law*. Perhaps his unique idea is that organisms are responsible for producing non-equilibrium, not the other way round [92], p. 1

“The main point of Bauer’s concept is not the non-equilibrium, but the function of organism producing the non-equilibrium, the capacity for self-adaptation, and the power for changing its functions in such a way that the system always gets the state of non-equilibrium always anew.”

Igamberdiev [94] is clear about the fundamental difference between Bauer and later pioneers of self-organizing systems, who base their ideas on the *thermodynamics* of irreversible processes, including Ilya Prigogine, who introduced this field, and later complexity theorists such as Herman Haken, who introduced the *synergetics framework*.

Although Prigogine’s and Haken’s frameworks provide theoretical foundations for describing dynamic complex systems, they fail to capture the specificities of *biological autonomy*. Igamberdiev argues that, mainly because they do not include the Aristotelian internal efficient causes intrinsic to the *phenomenon of life*, these frameworks are heuristically useful yet epistemologically inadequate for dealing with autopoietic living systems. I am not quite sure whether this is true, in part, regarding Haken, as I will later show that, in his “second foundation of synergetics,” he showed some respect for “top-down” causality and the circular interrelatedness of collective synergistic emergent properties with the lower-level components that produce them. Ultimately, Prigogine’s theory serves as a foundational root that leads to more specific theories such as Haken’s synergetics and Holland’s and Gell-Mann’s complex adaptive systems.

Bauer’s ideas are still influential and inspire theorists such as George E. Mikhailovsky, who has thoughtfully reexamined Bauer’s concepts and, drawing on them, has crafted an intriguing perspective that positions living organisms primarily as *chemodynamic systems*, surpassing the traditional view of them as mere thermodynamic entities. He introduced three fundamental laws of biochemodynamics, defined [95], pp. 14–16:

“As the study of the dynamics of biochemical reactions in living systems constrained and determined by the flows of energy and matter in these systems”, upon which the essential features of life can be derived.

Now we will turn our attention to the pioneer of non-equilibrium thermodynamics of irreversible processes, Ilya Prigogine, and the impact of his work for todays science of complexity. The concept of *dissipative structures* was introduced by the Russian-Belgian Nobel laureate Ilya Prigogine to explain *complex ordered systems* that self-organize by expelling entropy through continuous exchange of energy and matter with their environment, thereby increasing order and organization [96,97,98]. Indeed, if we seek the most decisive moment in the history of complexity science, it would be the formulation of the second law of thermodynamics, and then its uses to search for the meaning of life. This was the guiding light of Prigogine’s work.

In a Nobel lecture delivered in December 1977, later published in Science and entitled *Time, Structure and Fluctuations* [97], p. 777, Prigogine highlights many other revolutionary developments in physics stemming from *the second law of thermodynamics*, including Boltzmann’s work in kinetic theory, Planck’s discovery of quantum theory, and Einstein’s theory of spontaneous emission. Yet its role in explaining life’s *self-organization* and evolution remains particularly peculiar after so many years, inspiring countless frameworks and theories on the origin and evolution of life. Guided by the question of “how order in time and space spontaneously arises in chemical and biological systems” [99], Prigogine’s pioneering contributions to the mathematical and computational study of *emergence*, micro-to-macro transitions, and oscillatory dynamics of physiological networks are of immense significance, inspiring whole new fields such as the *Brusselator* and *Oregonator* models [99]. What is the difference between isolated microscopically reversible physical systems and dissipative systems? Well, the former tend, over time, to acquire *maximal entropy* and *maximal disorder*, while the latter, as being microscopically irreversible and open, interact with their environment to evolve from “disordered” to more “ordered” states and exhibit a complex structure, such as that of biological systems [100].

In reference to biological systems, Toussaint and Schneider [101], p. 3 argue that “similar processes, constrained by the second law of thermodynamics, give rise to the emergence of structure and process in a broad class of dissipative systems”. This means that in any system moved away from equilibrium (e.g., a biological system), where dynamic or kinetic conditions are satisfied, the system organizes itself to reduce the effect of the applied gradient during developmental phases (especially during early embryogenesis) (ibid.):

“Biosystems increase their total dissipation, develop more complex structures with greater energy flow, increase their cycling activity, develop greater diversity, and generate more hierarchical levels.”

It is difficulty to dissect the mechanism by which biological system as dissipative systems self-organized. Therefore, simple yet general mathematical and computational models, such as *cellular automata*, have been developed to describe irreversible “dissipative behavior” across a variety of physical, chemical, biological, and other systems [100].

According to Wolfram [100], p. 603, *cellular automata* represent physical systems in which both space and time are discrete, and physical quantities can assume only a limited set of distinct values. A *cellular automaton* is made of a regular, uniform lattice (or “array”), typically infinite in size, with each location referred to as a “cell.”

The state of a cellular automaton is determined entirely by the values of the variables at each cell. It evolves in discrete time steps, with the value of a variable at each cell influenced by the values of the variables in its “neighbourhood” during the previous time step. The “neighbourhood” of a cell usually includes the cell itself and all immediately adjacent cells. The values are updated “synchronously” based on the values of the variables in their neighbourhood from the preceding time step, according to a specific set of “local rules.”

From the early days, scientists realized the great potential of *cellular automata* to study the complexity patterns arising from simple, identical components capable of synergistic collective interactions, such as the growth of pigmentation patterns in mollusc shells [102]. Recent application of cellular automata by the Adamatzky group [103] to fungal cells allowed authors to devise “Elementary fungal cellular automata (EFCA)” and “Majority fungal automata” (MFA) to simulate the complexity of a fungal hyphae, which are separated by internal walls (septa). This septum possesses tiny pores that allow cytoplasm to flow between cells in a regulated manner. In other words, the cells can block this flow by forming Woronin bodies in an emergency to prevent cytoplasmic flow.

This particular *cellular automaton*, which they devised for (MFA), includes Woronin bodies in its cell-state transition rules. This automaton setup allows them to analyze “how the 256 elementary cellular automata rules are affected by the activation of Wb in different modes, increasing the complexity of the applied rule in some cases” [103], p. 341. According to the reviewer of this paper, many *cellular automata* models have already shown that the classical notions of co-equality between determinism and prediction are decoupled—although the system is completely determined, one must still compute the future.

Furthermore, in biology, there are countless examples of *dissipative structures* that change over time. One class of examples concerns biological rhythms, such as cell excitability, circadian rhythms, the *cell cycle clock*, and cellular rhythms involving the opposing roles of the proteins p53 and NF-κB in cancer [99]. According to Goldbeter [99], many discoveries and models in biology, chemistry, and ecology during the 1950s and 1960s inspired Prigogine’s theory. Some of these include the Lotka–Volterra model of oscillations in predator–prey systems and the Hodgkin–Huxley model of single-neuron action potential generation and propagation, which connects the electrochemical properties of membrane ion channels (Na+ and K+ ion channels) and the transporter (Na, K-ATPase, which forms the electrochemical gradient across membranes) with the generation and propagation of electrical signals in excitable cells.

### 3.2. The Second Root: Belousov–Zhabotinsky Reaction, Brusselator and Oregonator Models

Nonlinear oscillatory dynamics and the Belousov–Zhabotinsky (BZ) reaction connect the first and second roots. The second root is considered by some, such as Siegfried Roth [104], as the earliest and most direct for the history of biological pattern formation theory, dating back to the first half of the 20th century and including the search for the chemical basis of pattern formation, culminating in Alan Turing’s [105] attempts to explain the role of genes during embryogenesis, with reference to the chemical and mechanical properties of the cell. In biochemistry, the discovery of what became known as the BZ reaction, which exhibits spatiotemporal oscillatory dynamics, was quintessential in efforts to explain oscillatory dynamics in cells and organisms, according to Goldbeter [99]. Indeed, Boris Belousov, who was studying the oxidation of citric acid, made a significant discovery that reverberates today more than ever across the systems cell biology paradigm in studies of biochemical clocks, cellular decision-making, and signalling networks in time and space [106].

Tyson [106], pp. 185–186 explains the historical context of Belousov’s discovery: By the late 1940s, it was known that mitochondria, as they oxidised acetic acid (CH_3_COOH) to CO_2_, generated ATP by a “mysterious” process involving cytochromes—redox-active proteins employing Fe^2+^/Fe^3+^ ions to shuttle electrons from donors to receptors. Around 1950, Belousov, eager to understand how transition metal ions catalysed the oxidation of di- and tricarboxylic acids, focused on the oxidation of citric acid by bromate ions in acidic solution, with cerium ions (Ce^3+^/Ce^4+^) as a catalyst. He made a surprising discovery: under certain reactant concentrations, the chemical solution oscillated repeatedly between clear (Ce^3+^) and pale yellow (Ce^4+^). In other words, he discovered nonlinear oscillatory dynamics (a nonlinear oscillator) in an in vitro system relevant to biological in vivo systems such as cells. Ilya Prigogine called this reaction the most important discovery of the 20th century [107]. Nowadays, the BZ reaction, as a functional model of biological phenomena, is used by biologists, physicists, and chemists to model a variety of biological systems and processes [108].

However, recognition of the nonlinear, autocatalytic oscillatory potential of the BZ reaction came in 1984, when the oscillatory chemical reactions (OCRs) model, which challenges *the second law of thermodynamics* regarding the BZ reaction’s oscillatory behavior, was proposed and developed [109]. Before that, Field, Körös, and Noyes (FKN) were the first to propose a realistic mechanism to explain the temporal oscillation of the BZ reaction, later dubbed the *Oregonator* [109,110]. The FKN model proposed three subprocesses and three controlling factors, each governing the kinetics of the entire reaction, specifically the concentrations of bromide and cerium ions [109]. According to Gupta et al. [109], the *Oregonator* model is a five-step version of the FKN mechanism: it includes five coupled elementary reactions and only three independent chemical intermediates that summarise the main features of the BZ reaction. The *Brusselator*, proposed by Prigogine and Lefever in 1968 in Brussels, consists of four elementary reactions and was the first attempt to model the BZ reaction [111,112,113].

Walter Fontana and Leo Buss proposed a minimal theory of biological organisation based on chemistry and mathematics (λ-calculus), capable of simulating and capturing [114], p. 1; [115]: (1) the constructive feature of chemistry, where the collision of molecules generates specific new molecules, and (2) chemistry’s diversity of equivalence classes, where many different reactants can yield the same stable product.

Recently, researchers have begun using AI models to simulate and explain the conditions and processes involved in the transition from molecular interactions to the emergence of self-organizing structures. For example, using the AlChemy model—an artificial chemistry model based on λ-calculus—Mathis et al. [115] showed that simple computational rules can give rise to stable, complex, self-organizing emergent structures.

This and other artificial chemistry models are important tools for attempts to create artificial life, engineer synthetic biological life (cells), or understand the conditions and processes necessary for transitioning from bioinorganic molecules (e.g., proteins, enzymes, and DNA containing inorganic components such as six relatively light elements, affectionately called CHONPS, and cofactor ions such as Cu^2+^, Mg^2+^, or Fe^2+^) into the first self-organised replicating structures capable of supporting metabolism [116,117]. The key role in establishing these self-reproducing metabolisms is played by an autocatalyzed subset of Turing-complete reactions, which can be simulated by a minimalistic artificial chemistry with conservation laws [118]. A single run of this chemistry, with no external intervention, generates different emergent structures, including ones that self-reproduce in each cycle; these emergent structures, in the form of recursive algorithms, acquire basic constituents from the environment and decompose them, mimicking a biological metabolism [118].

BZ reactions and models based on them to simulate autocatalytic sets and subsets now account for many diverse biological processes, such as metabolism, signalling, and cell development, whose oscillatory dynamics control important features of cell physiology, including DNA synthesis, glycolysis, cyclic AMP production, protein-interaction networks in the eukaryotic cell cycle, and many more [119]. Combined with Prigogine’s *dissipative structures*, *Brusselators* and *Oregonators* provide firm roots for self-organization in biology, about which much remains to be discovered.

### 3.3. The Third Root: Autopoiesis

Furthermore, the following considerations will be useful for illustrating the third root. In the 1970s, Chilean biologists Francisco Varela and Humberto Maturana succeeded in uniting previously scattered research involving Prigogine’s *dissipative structures*, order by fluctuations, Eigen and Schuster’s theory of self-organizing hypercycles of catalytic synthesis of complex nucleic acids and proteins, and the spontaneous social orders of von Hayek, under the single framework called the theory of *autopoiesis* (self-production) [17,18,19,20,21]. In the spirit of Bauer’s and Prigogine’s work, Varela and Maturana take “order” as foundational for any complex system. The same organization can be constructed with different components [20], p. 7:

“The same organization may be realized in different systems with different kinds of components as long as these components have the properties which realize the required relations.”

They clearly refer here to the multiple realizability of the same organization with different components, but I am not sure they also mean the multiple realizability of the same properties by different components. For example, it is difficult to argue that the properties of water, produced by hydrogen and oxygen, could be reproduced by other chemical components. However, they take from this that some basic universal principles govern organization across different systems, from machines to living beings. These principles are as follows [20], p. 8:

“Autopoietic organization of life is defined as unity by a network of productions of components which (1) participate recursively in the same network of productions of components which produced these components (closure in production), and (2) realize the network of productions as a unity in the space in which the components exist (closure in space).”

They provide a striking example from cell biology (ibid.):

“In the case of a cell, it is a network of chemical reactions which produce molecules such that (1) through their interactions generate and participate recursively in the same network of reactions which produced them, and (2) realize the cell as a material unity.”

These *principles of organization* allow the cell, isolated from the background topographically and operationally, to maintain its organization despite the continuous turnover of matter. Despite changes in the form and specificity of its constitutive chemical reactions, the organization persists [20]. Simply stated, organisms are characterized or defined by *organizational closure*. They even make an important distinction between autopoiesis and alopoiesis. In contrast to living systems, mechanistic systems do not produce components or processes themselves. Humans produce these components and assemble them. However, we must be careful here. In today’s robotics, artificial intelligence, and machine learning, although these machines are not self-producing, they exhibit a certain degree of autonomy and self-learning [120].

Although successful in theoretical biology and in the fields of artificial life and the origins of life, *autopoiesis* was not well received by mainstream biologists, mainly because deriving specific, testable hypotheses has been challenging [86]. However, it has recently undergone significant conceptual reappropriation in the so-called *enactivist* and *agential approaches* to life and mind, to operationalize concepts related to self-individuation such as precariousness (life-or-death stakes), *adaptivity*, and *agency* [86,121,122]. Here, *self-individuation* means the system actively takes steps to preserve its identity, whether at the cellular or the mental level. In addition to *organizational closure*, the embodiment of an objective function that provides a ‘goal’ should be recognized as foundational to self-organized biological systems [123].

In my view, autopoiesis, like other theories within the self-organization framework over the past few decades, has struggled to address the “top-down” effects of the environment on organisms, except perhaps Haken’s synergetics. Although many argue that it allows for the transfer of matter and energy from an organism’s surroundings, the role of epigenetics must be considered. However, recent advances in genetics are straightforward: epigenetic mechanisms influenced by environmental factors can directly regulate genetic processes via DNA methylation and microRNA expression [124,125]. These changes can lead to variations in phenotype, prompting a dialogue between Neo-Lamarckism and Neo-Darwinism [125]. In 1802, Jean-Baptiste Lamarck (Lamarckian theory of evolution) proposed that the environment could directly modify phenotype in a heritable way, challenging the Darwinian and later neo-Darwinian perspectives that natural selection acts on genetic alterations and random mutations that drive phenotypic variation [125]. In Darwinian worldviews, a phenotypic trait resulting from a mutation is fixed in the population only when it is proven to increase fitness (survivability and reproduction) [126].

Besides the *enactivist* and *agential* upgrade of *autopoiesis*, what other improvements has *autopoiesis* undergone? Evident in many accounts is the reference to Aristotle and the attribution of his four causes to the autopoietic, self-organized biological system. In the context of Aristotle’s metaphysics, organisms are closely associated with efficient causation (the agency and change in processes within the system), as they produce their own catalysts [127]. Simultaneously, they are close to *material causation* (the constituents of which the system is composed) due to a net, overall irreversible process that provides a thermodynamic driving force for metabolism [127]. In other words, *efficient cause* remains constant. At the same time, the regulatory processes that maintain the organization (a material-independent property) persist as long as the organism is alive [128]. However, their *material components* (*material causation*) constantly change as these regulatory components are synthesized and degraded to maintain homeostasis [128].

Moreover, over the last few decades, *self-organization* and *autopoiesis* in biology have been reframed to include understanding that *constraints* have a causal role. Noble [87] and Ellis [88] argue that the environment can influence biological processes at all levels. Higher levels can constrain and shape lower levels, thereby providing a rationale for “top-down” causality. As a result, “bottom-up” genetic level (e.g., DNA to proteins) is constrained by higher levels in a context-sensitive manner. This provides an arena for the “meshing” of “bottom-up” molecular interactions and “top-down” *constraints*, and integration of functions across multiple levels, such as the molecular, cellular, and tissue or organ levels. It is important to note that “top-down” effects are not limited to biology. In chemistry, the collections of molecules can take a thermodynamically stable emergent conformation that differs from that of individual molecules. Additionally, these molecular aggregates can influence the structure and behaviour of the individual molecules within them, suggesting both emergent properties and “top-down” causality [129].

Some philosophers of science, such as Ross [54], argue that, unlike the *standard model*, which denies a causal role for *constraints*, there is considerable evidence to correct this misconception and to ascribe a causal status to *constraints* in biology. She identifies two types of *causal constraints*. The first type involves changes in the *spatial location* (e.g., anatomical) of an entity over time, such as blood vessels that regulate blood flow, nerve tracts that control the flow of nerve signals, and lymph vessels that restrict the spread of cancer.

The second type of *causal constraint* concerns the causal effects of *constraints* on the composition of a system’s constituents (e.g., metabolic pathways, stem cell pathways, and developmental pathways). According to Ross [54], these *constraints* play a crucial role in directing the final product of, for instance, metabolic pathways; that is, they specify which ‘types of downstream products the upstream substrate is converted into’. Lehman and Kauffman [130] argue that *boundary conditions* are the driving force behind the constraint-closed system. In other words, these conditions provide *constraints* for a system. Stuart Kauffman and Andrea Roli [131], p. 7 propose that living organisms are Kantian in nature. They are “open, thermodynamic, self-reproducing chemical reaction networks that are Kantian wholes that achieve *catalytic closure*, *constraint closure*, and are spatially bounded, or enclosed, often by an enclosing lipid membrane (spatial closure)”.

In Kauffman and Roli’s model *Catalytic closure* refers to the molecular-enzyme component that exists for and by means of the entire set of other peptides in a metabolic pathway. Accordingly, *constraint closure*, by contrast, emphasizes that a complex system, such as a living cell, “literally constructs itself by doing thermodynamic work to construct the boundary conditions that constrain the release of energy to construct the very same boundary conditions” (ibid.). This *constraint closure* arises from non-equilibrium processes, characterized by the “work produced as a constrained release of energy” [131].

*Spatial closure*, on the other hand, means that living systems are to some degree isolated from the environment, often described as a compartmentalized organization of cells in which metabolic processes occur within the lipid membrane [131]. These *two closures* are key to unlocking a new frontier in science: the emergence of life as a novel property of a prebiotically assembled system, and their integration establishes a permanent *system–process duality* [132]. This duality, defended by Gómez-Márquez, renders the organization and functioning of molecular and cellular networks inseparable, a discovery that has renewed excitement in the field of biochemistry. The operative integration of these *two closures* is also prompted by Faggian [133], p. 1:

“When the components of a system meet Closure conditions, they constrain the existence of one another over a time interval. Each element becomes both a constraint on and a product of other *constraints*, creating a network of interdependencies that drive the system’s organization.”

### 3.4. The Fourth Root: Systems Biology

The fourth root of *self-organization* in biology is systems biology. As François Jacob [134] famously pointed out, much of the history of biology and biological thought in the nineteenth and twentieth centuries lies at the intersection of *reductionism* and *holism*. Taking historical context into account, the declining significance of reductionist approaches in biology paved the way for a resurgence of holistic perspectives (see [135,136]). Before that, the mid-twentieth-century discovery of the structure of DNA by Watson and Crick in 1953, the molecule that carries and transmits genetic information, led to the accelerated development of molecular biology, fostering the belief that the secrets of life could be uncovered and explained at the most fundamental molecular-genetic level [137]. There was little room for holism and holistic thinking in those days. However, everything began to tilt in favour of *holism* at the end of the twentieth century when the sequencing of the human genome, as part of the *Human Genome Project*, was announced. Conceptually, systems biology emerges from this conflict, in which *holism* prevailed over *reductionism* (see [138,139]).

The post-genome era, as many call it, marked a turning point in the rise of systems biology and a new understanding of organisms as holistic systems, for which scientists needed deeper characterization and understanding, leading them to develop new approaches and methods. This newly formed systems biology, shaped by large-scale high-throughput molecular data (systems molecular biology or pragmatic systems biology), alongside insights from *non-equilibrium thermodynamics* and modeling (systems-theoretical biology), introduced a new way of thinking about molecular data, biological hierarchical organization, the role of the gene, “top-down” causation, modelling, and, above all, how to pursue the explanation of complex biological systems holistically [138,139,140,141]. It also introduced a revised definition of the gene, which now includes not only a sequence of DNA but also the complex multilevel processes of modification and interactions between gene products (RNA and proteins), other genes, and the environment that ultimately produce phenotypes (genetic characteristics) in a controllable, constrained manner [142]. This was wisely captured by Denis Noble [87], p. 60:

“A difference in DNA sequence may have a wide variety of possible phenotypic effects, including no effect at all, until the boundary conditions are set, including the actions of many other genes, the metabolic and other states of the cell or organism, and the environment in which the organism exists.”

The concept of *constraints* suggests that higher levels restrict bottom-up molecular events. In open, complex biological systems, these *constraints* influence nonlinear, synergetic interactions at lower molecular, cellular, and genetic levels to maintain dynamic, emergent patterning within the organism.

### 3.5. The Fifth Root: Self-Organization and Information

This system-related understanding of the transmission of genetic information provides a rationale for the last, but not least, root of *self-organization* in biology. Although at first sight biological information at the molecular level appears linear (see [143]), errors and noise arising from continuous interactions between the genome and the environment (molecular oscillators) lead to large-scale nonlinear information processing in cells and organisms (large-scale biological patterns) (see [144,145]). In principle, biological systems function by maintaining their state far from equilibrium with their environment. To sustain this continuous nonlinear operation, organisms must control all mechanisms responsible for its production in an integrated manner [146,147]. This control involves mechanisms that sense and respond to changes in conditions within the organism or its environment, serving as *constraints* that act on other mechanisms to adjust their operation across scales [147]. Based on what has been said so far, it is clear that any reference to *self-organization*, including theories, frameworks, and their operationalization, cannot be considered in isolation, as they all seem interwoven and interconnected.

Haken’s second foundation of his theory of *synergetics* best illustrates the union between the *self-organization* and *information* in biology and elsewhere. While the first foundation of *synergetics* dealt with quantitative and formal descriptions of lower-level components that produce a macroscopic holistic system, the second foundation addresses the meaningful production, extraction, and exchange of information by or within complex systems that evolve spontaneously, i.e., by self-organization—“a means by which the system and its parts extract, or produce meaning and/or action from signals” [66], p. 4.

Haken and Portugali use the concept of Shannon’s information, which is capable of addressing the following aspects of a message: (1) irrespective of its *meaning* (syntactic); (2) *semantic* and *pragmatic* forms of *information*, which deal with the meaning conveyed by messages; and (3) *information adaptation*, which refers to the interplay between *Shannon’s information* and *semantic* or *pragmatic information*.

Without delving deeply into it, *information*, *syntax*, and *meaning* (semantics) are closely connected and essential for self-organized complex systems. For example, biologist Howard Pattee, motivated to understand the organism from within, uses explicitly linguistic terms and concepts to address what he identifies as the cellular “semantic closure” and “epistemic cut”. More specifically, “semantic closure” refers to “the self-referential mechanism through which symbols actively construct and interpret their own functional contexts” [148].

Messages and codes of living organisms are meaningful only within the natural systems—such as organisms, ecosystems, and so on—in which this information is encoded and used, “acquiring their ‘meaning’” (always operational and only operationally discernible for Pattee) within a system, organism, ecosystem, etc. [149], p. 161. On this view, *information* and its *meaning* are intrinsically linked to organisms, as they actively acquire, process, and use information about their internal and external environments to sustain their physiology and behaviour [150]. I will not discuss *self-organization* further in the informational context, except perhaps for references to information found in theories discussed in this paper, as it warrants much deeper and separate treatment, especially considering ongoing efforts to connect *emergence* and *information* (see [151]).

Taken together, the thermodynamic, physicochemical, systems-autopoietic, and informational roots of self-organized biological systems make it clear that science currently lacks a unified and comprehensive framework in biology, let alone in the broader natural and socio-technical sciences. Although there are many theories of *self-organization* with different axiomatic foundations and computational formulations, often associated with their originators (e.g., Bak’s self-organized criticality, Haken’s synergetics), they initially address only some of these roots. However, as these theories and the science have evolved, it is evident that some have undergone further development, such as Haken’s *synergetics*, to incorporate advances unavailable at the time they were proposed. The proposed historical classification of roots, which is largely static, only partially reflects the historical patterns that have led to current self-organization theories in biology, which, after all, show significant convergence and complementarity.

## 4. Theories of Self-Organization: All Paths Lead to Emergence and Holism

As discussed, the *self-organization* framework in biology, developed in the twentieth century, has clarified the universal principles governing *self-organization*, order, and control in biological systems. However, the specific ways in which thermodynamic principles operate within these systems, according to certain compatible rules, remain unclear. This has led to the development of various theories of *self-organization*, which both inspire the imagination and drive the practical work of biologists. Each theory has its strengths and limitations, and it is important for the reader to critically assess their apparent and hidden value for further study and modeling of biological systems. Due to constraints, the following will offer only a brief conceptual overview, not a mathematical or computational one, of these theories and their relevance to real-life biological problems.

### 4.1. Self-Organized Criticality and Complex Adaptive Theory

Understanding self-organized criticality (SOC), complex adaptive systems (CAS), and self-organization in general requires attention to the concept of a “strange attractor,” a key feature of dissipative chaotic systems, originally proposed by David Ruelle and Floris Takens in their 1971 paper on the nature of turbulence [152]. They introduced a mathematical model of a physical system consisting of a viscous fluid and rigid bodies under varying conditions, which takes the system out of *steady state*. Variations in factors such as pumping and heating lead to one of the following results [152], p. 167: (a) the fluid motion may remain steady but change its symmetry pattern; (b) the fluid motion may become periodic in time; (c) for sufficiently large μ, the fluid motion becomes very complicated, irregular, and chaotic, resulting in turbulence. Here, μ depends on the situation and can be the Reynolds number, the Rayleigh number, etc. I recommend their paper for details about the mathematics behind their model.

Ruelle and Takens’ model showed how the nonlinear motion of fluids can produce chaotic turbulence and “strange attractors.” More broadly, a *strange attractor* in dynamical systems is a type of attractor—a region or shape to which points are “pulled” as the result of a certain process—that arises in certain nonlinear systems and is characterized by its fractal structure (https://www.dynamicmath.xyz/strange-attractors/, accessed on 31 March 2026). In other words, *attractors* are long-term fractal geometric structures in phase space characterized by steady states toward which a dynamical system evolves. While trajectories within these *attractors* appear unpredictable, the attractors themselves are orderly, unchanging, and elegant geometric structures (https://annex.exploratorium.edu, accessed on 31 March 2026).

*Attractors* were later explored and appropriated in biology (*bioattractors*, *epigenetic attractors*) by Brian C. Goodwin and Peter T. Saunders in their book Theoretical Biology: Epigenetic and Evolutionary Order from Complex Systems [153], where they discuss how self-organizing processes drive organismal complexity beyond the Neo-Darwinian paradigm of evolution focused on genes and natural selection. More specifically, self-organizing dynamic processes lead to *stable states*, called “attractors” in an *epigenetic landscape*, a concept proposed by Conrad Waddington in the 1940s, especially in his book *The Strategy of the Genes* [154]. One example will clarify this.

Building on the *epigenetic landscape* with insights from *chaos theory*, Jaeger and Monk [155], p. 2270 provide the following example to substantiate what Davila-Velderrain et al. [156] call the *Epigenetic Attractors Landscape* in the context of gene regulatory networks and dynamical systems theory: In the case of a specific genotype and specific environmental conditions, and in a population of individuals with variation in initial conditions (due to the environment) and system parameters (due to genetic variation), the system will follow a particular trajectory to produce a particular phenotypic outcome. Any combination of initial regulator concentrations (transcription factors) serves as the starting point from which the system’s equations determine a dynamic trajectory by describing the rate of change in the system state over time (the temporal progression of the system). Jaeger and Monk define the totality of possible trajectories in *phase space* as the flow of the system, and these trajectories converge toward subregions of *phase space* called “attractors,” which are the stable steady states of the system that can be described by corresponding fractal dimensions. Notably, “each *attractor* has an associated region of *phase space*—called its *basin of attraction*—that contains the set of trajectories that converge toward it” (ibid.). The *attractors*, their associated *basins*, and their *bifurcations* are defining features of the regulatory and evolutionary potential of a biological system, Jaeger and Monk argue.

To track the entropy production along the unpredictable trajectories that lead to an *attractor*, a *fluctuation theorem* has been proposed, which accounts for the statistical rule governing entropy production not only at the level of the organism but also at the population-ecological level of life’s organization and functionality [157], p. 224. The *fluctuation theorem*, with the following *directionality principles* for demographic stability, underscores the general significance of entropy for demographic and ecological studies (ibid.):

(1) A unidirectional increase in stability in populations subject to bounded growth constraints,
(2) A unidirectional decrease in stability in large populations subject to unbounded growth constraints,
(3) Random, non-directional changes in stability in small populations subject to unbounded growth constraints.

I mentioned fractals in association with attractors on multiple ocassions, but what are they? Fractals, introduced by Benoit Mandelbrot in his book *Les Objets Fractals, Forme, Hasard et Dimension* in the 1970s, are infinitely complex patterns that maintain self-similarity across scales [158,159]. *Fractals* are, at least approximately, self-similar shapes consisting of reduced versions of themselves, but are too irregular to be described by Euclidean geometry [160]. As pointed out by Mandelbrot [158], fractal geometry represents a geometric framework occupying a middle ground between the excessive geometric order of Euclid and the geometric chaos of general mathematics.

Fractal dimension (FD), an essential feature of *fractals*, is an important measure that quantifies the complexity of a fractal or complex system, or better say, the inherent irregularity of a fractal object by a number [161]. It provides insight into the amount of space a fractal occupies and is used to measure the density with which a fractal fills space, that is, how many new details appear as the resolution increases [161]. FD is not an integer and is usually larger than the Euclidean dimension, providing information about their geometric structure at different scales [162].

*Self-avoiding walks* and *space-filling curves* with integer dimensions are important for understanding topographical *self-organization*. According to Google AI:

“A space-filling curves represent two extremes of fractal path behaviour, where the former generally has a fractal dimension less than the embedding space, and the latter fills the space completely.”

An earlier numerical study of *self-avoiding walks* by Havlin and ben-Avraham [163] found that, for polymers (e.g., Proteins, DNA), a single chain possesses a well-defined fractal dimensionality, i.e., it exhibits statistical self-similarity. However, as the number of steps in the Monte Carlo simulation increases, the fluctuations set in, and the fractal dimensionality measured on a single-chain configuration vanishes. Similarly, by building on Paul Flory’s insight that *self-avoiding walks* (i.e., visiting every vertex at most once) on a lattice can model polymer chains, Duminil-Copin et al. [164] showed mathematically that these supercritical self-avoiding walks are space-filling. For example, this may be an important process in protein folding or DNA packing in the cell, which must occur in a way that fills volume without self-intersection to ensure genome stability and functionality. If the chains of these molecules intersected, it would be impossible, for example, for transcription and replication processes to occur in DNA (see [165]) for DNA damage and repair.

In a map-based approach to *exploratory behavior*, which supposes that navigation towards a goal is guided by the subject’s knowledge of the environment (i.e., allocentric navigation), animals and humans can be linked to map learning [166]. For instance, rats can use cognitive maps acquired either by using a “base familiar point” or by observing the success or failure of other individuals to explore paths in the environment that may lead them to shelter, food, a mating partner, or to avoid obstacles [167,168]. In other words, they could topographically explore more new territory rather than just circling around the familiar area. For example, Yamamoto et al. [167], p. 99 showed that the *exploratory behaviour* of thirsty rats systematically progressed to areas distant from the starting location in an unfamiliar environment; this anchor point, or “base of operations,” allows the formation of a network of topographic relations among objects in particular locales. Thus, rats fill more space while avoiding self-intersection. However, this is just one possibility of how they behave in nature or captivity, as they may, due to anxiety, choose not to explore new areas and instead remain in the familiar ones.

Mandelbrot also proposed *multifractals*, characterized by a continuous spectrum of fractal dimensions, to describe phenomena in which the distribution of quantities is self-similar across many scales [169]. Mathematical reasons for the existence of *multifractals* include the presence of *multiplicative iterative schemes*, distinct from additive ones [170]. In practice, the multiplicative data-generating mechanisms and methods based on them seem better able to capture time-to-event outcomes, such as survival rates, in analyses of real data from controlled randomised trials in biomedicine than additive mechanisms or schemes [171]. Such a trial is an experimental setup designed to evaluate the efficacy or safety of an intervention, or the effects of environmental hazards or differing lifestyles, by minimizing bias through random selection of study participants [172].

Now, let us discuss self-organized criticality (SOC). SOC holds that the system evolves toward a stable, orderly state even after being driven far from equilibrium [173]. This should account for the complex patterns observed in widespread 1/f noise, power scaling, and self-similar fractal structures found in nature [173]. Essentially, this means that biological systems and other natural dynamic entities with spatial degrees of freedom, poised between order and disorder, can reach a self-organized critical point, generating complex, self-similar, fractal-like structures in both space and time [153,173]. Bak et al. [173] provide a paradigmatic example of a sandpile to illustrate this theory also discussed in [174], pp. 181–182. When too many grains are added, disruption of local stability may occur as the “unstable site relaxes by distributing grains to the neighbour site, which may induce more instabilities and a cascade of events (or an avalanche)”. Tadic and Melnik [174], pp. 181–182 note that the avalanche propagates until all sites are stable again, at the expense of some grains leaving the system, thus maintaining stable fluctuations in the total mass. Most importantly, as they state (ibid.):

“The driving time scale is much slower than the avalanche propagation, and the avalanche size is not linearly correlated with the driving force. The multi-scale response is characterised by self-similarity and scaling. Relevant quantities in the SOC state have power-law behaviour, fractal geometry, and scale invariance.”

The SOC framework appears to have the potential to unify “the origins of the power-law behaviour observed in different complex systems” [175], p. 43. On the other hand, SOC states emerge in many open systems at different scales and types of interactions, such as “interacting nonlinear systems with many constitutive parts”, constraints (“the substrate geometry allowing interactions and communication paths”), and cooperation (“interactions among the system’s elements at different scales”) [174,176]. According to Tadić and Melnik [174], this drives increased structural complexity, further supporting complex dynamical behaviours. The example from biology, such as the pattern of metabolic connectivity in the context of SOC, will be discussed in the following Section 3.3.

However, the concept of SOC has been criticized by *Complex Adaptive Systems* (CAS) theorists, mainly because SOC fails to include the phenomena of adaptation and learning, i.e., the fact that many components adapt and learn as they interact in complex systems [31,177]. The main shortcoming of SOC lies in its mathematical methods. Techniques such as fixed points and attractors, developed within this framework, do not adequately address agents’ learning and adaptation processes [177]. According to CAS, introduced by Gell-Mann [178] and Holland [177,179,180], many complex systems share fundamental commonalities and general principles, even if they appear very different at first glance [34,177,180]. The central premise of CAS is that a complex system is composed of numerous interacting adaptive agents capable of learning [177,178,179,180]. What characteristics of agents capable of learning does SOC omit?

According to John Holland [177], pp. 1–2, a pioneer in *Complex Adaptive Systems* (CAS), the following features of this theory—*parallelism*, *conditional action*, *modularity, adaptation*, and *evolution*—are supported by concrete physical evidence, particularly from biology. The first characteristic is *Parallelism*.

(1) CAS comprises numerous agents that interact by sending and receiving signals. These agents operate simultaneously, generating a large volume of signals.

Holland illustrates this feature with an example from biology: proteins act as signalling molecules within cells, forming signalling pathways. Many proteins must interact in a highly coordinated manner to ensure the cell functions properly. They operate in reaction cascades and cycles, providing both positive and negative feedback to other cascades and cycles within the cell. The second feature is *Conditional Actions*:

(2) Agents in a complex system adjust their behavior in response to signals from their surroundings. This means that agents operate according to an IF/THEN structure (conditional statements or implications, p → q): IF [signal vector x is present], THEN [execute action y]. The action may then serve as a signal itself, creating complex feedback loops, or it may result in a visible action within the agent’s environment.

In microbiology, agent-based modeling (ABM) is widely used to study microbial communities [181]. ABMs often focus on molecular events, single-cell behaviours, cellular interactions, and cell-environment coupling. As described by Nagarajan et al. [181], p. 3565, molecular events include gene regulation, metabolic reactions, and signal transduction. Single-cell processes involve cellular growth, division, and chemotactic migration in response to attractant gradients. Cellular interactions may be mechanical, resulting from forces exerted by neighbouring cells, or chemical, arising from the secretion of toxins or the sharing of resources. These biotic interactions occur between individuals of the same species, different species, or even entirely different genera, families, or domains [182]. Interactions among cells in a microbial community are often nonlinear and occur when a specific threshold is reached [183]. Nutrient uptake from the surroundings and biomass dispersal driven by fluid flow are examples of cell-environment coupling [181]. Abiotic factors, such as surface type (e.g., bark), temperature changes, nutrient availability, and pressure variations, also play a significant role in short- and long-term changes in microbial communities [184].

In terms of real life example, microorganisms (bacteria, archaea, fungi, microalgae, and viruses) and their complex microbial communities have been exposed to a wide range of environmental factors throughout their evolutionary history [183,185]. These factors can be categorized by their stability and variability: some remain relatively constant over long periods, such as geological epochs; others change slowly, like the general increase in annual temperatures; some fluctuate periodically, as in day-night cycles and seasonal variations; while others change frequently and somewhat unpredictably, such as nutrient loading [185]. These environmental changes occur over various timescales, from the lifespan of an individual cell to multiple generations [185]. In response to short-term environmental stimuli (environmental sensing), they reversibly adjust their physiological networks to both maximize resource utilization and maintain structural and genetic integrity (genetic and cellular repair mechanisms) [185,186]. Holland’s third feature is *Modularity*:

(3) In an agent, groups of rules often function as “subroutines.” For example, the agent can respond to various situations by executing a sequence of these rules. These “subroutines” act as building blocks that can be combined to address new and unexpected situations, rather than requiring a separate rule for every possible scenario. As these potentially useful building blocks are tested frequently in a wide range of contexts, their effectiveness is quickly validated or disproved.

He provides an example of the citric acid cycle to demonstrate *modularity*. The citric acid cycle (Krebs cycle) comprises eight proteins that interact to form a loop found in all aerobic organisms, from bacteria to elephants. In genetics, many examples of *modularity* can also be observed, providing the necessary robustness—the ability of a system to withstand disturbances and maintain functionality under changing environmental conditions [187,188]. For example, in prokaryotes, structurally similar sigma factors regulate distinct sets of genes. Under specific conditions, a dysfunctional sigma factor can be replaced by another sigma factor, enabling the organism to remain functional [189]. Furthermore, the same transcription factor that regulates a set of genes can form functional modules in *Pseudomonas aeruginosa*, which is important for adaptation and survival in challenging environments [190]. These examples are discussed in Alcalá-Corona [188].

Holland’s final feature of CAS theory is *Adaptation* and *Evolution*.

(4) The agents in a complex adaptive system evolve. These changes typically involve adaptations that enhance performance, rather than random variations. Adaptation involves addressing two key problems: the credit assignment problem and the rule discovery problem.

It is also worth noting that credit assignment means that “an agent’s performance is the result of an intricate skein of interactions extending over space and time” [177], p. 2. The rule discovery problem concerns situations in which one of an agent’s rules is ineffective or detrimental. However, their replacement is not ad hoc; rather, new rules to be assigned should be plausible in terms of the agent’s experience. These two criteria of adaptation and evolution relate to game theory, and I recommend Holland’s paper and van Bilsen et al. [191] for further insights into this theme. The biological example of the fourth aspect of CAS builds on Holland’s second feature. In microorganisms, when environmental changes occur repeatedly, signals are converted into and stored as molecular and genetic information [182,185]. This means that natural selection may favour signal-related changes in molecular and physiological networks that enhance fitness and reproductive success.

### 4.2. Agency

In simple terms, agency is the inherent ability of an organism to act independently and make choices or behavior “intrinsic to an organism and initiated by it” [192]. The idea of goal-directedness and purposeful behavior, in living beings, is not new and can be traced back to the Ancient Greeks. In the 18th century, the philosopher Immanuel Kant emphasized that both *teleology* and *mechanism* are essential for understanding the living world. Rama S. Singh [193], p. 258 has suggested that, given Kant’s insistence on both *teleology* and *mechanisms* to explain the behaviour of organisms, complementarity between the *constitutive* and *heuristic components* of *teleology* may provide a necessary reconciliation between *mechanistic-reductionist Newtonian mechanisms* and *finalistic* or *holistic biology*. In other words, according to Singh [193], the *constitutive component of teleology* addresses organisms’ purposive adaptive behaviors grounded in complexity and redundancy. At the same time, the *heuristic component* encompasses human cognition, including awareness of time, as well as anthropomorphic, predisposed, *goal-oriented behavior*.

In this context, “constitutive” refers to something *inherent* in the organism, while “heuristic” refers to the methods used to understand the organism’s goal-directed behavior. As vindicated by Georg Toepfer [194], p. 113 evolutionary theory cannot provide the foundation for teleology in biology which give rise to “identity crisis” because *teleological reasoning* is precious and much needed in specifying the identity of biological systems, i.e., “organization” or “the causal pattern of interdependence of parts with certain effects of each part being relevant for the working of the system.”

Due to centuries of ambiguity and inaccuracy surrounding the term “teleology” in biological explanations, especially since Kant (see [195]), biologists in the latter half of the 20th century began to adopt more precise terminology, such as “teleonomics”. This shift was largely influenced by the chronobiologist Colin Pittendrigh. The term “teleonomic” is appropriate for understanding true complexity [196], p. 607:

“Complexity involves vast amounts of stored information and hierarchically organized structures that process information purposefully, particularly through the implementation of goal-seeking feedback loops. This structure gives the appearance of purposeful behavior (i.e., ‘teleonomic’).”

Holland’s *minimal agential characteristics* might not be sufficient for biology. To make it relevant to biological systems that express *intrinsic goal-directedness*, Watson [197] proposes, in a holistic manner, that the “agency of a system can be more than the sum of the agency of its parts.” In fact, agency in biological frameworks can be viewed as an extension of CAS theory that more explicitly accounts for the behavioural uniqueness of biological organisms by focusing on the capacity of biological agents (organisms) to learn and adapt to achieve specific goals (such as resource allocation, mating, etc.). Indeed, according to DiFrisco and Gawne [198], p. 143, agency should be “understood as the capacity for goal-directed, self-determining activity—a capacity that is present in all organisms irrespective of their complexity and whether or not they have a nervous system.”

Rosslenbroich et al. [199] go further, proposing that agency, should be understood and explored as an *intrinsic*, or rather *immanent*, multilevel feature of living organisms, with the following gradation of agency levels: *basic life processes*, *the organismic level*, *the ontogenetic level*, *directed agency*, *directed agency with extended flexibility*, and a level that includes the *capacity to pursue preconceived goals*. Rosslenbroich et al. [199] also highlight the close relationship between *agency* and *biological autonomy*, as enhanced physiological and behavioural *autonomy* extends the range of self-generated behavioural possibilities, flexible actions, and reactions; *autonomy* through evolution coincides with higher levels of *agency*.

Although accused of lacking a proper supporting research programme [198], the agency appears to have an important place in contemporary evolutionary developmental biology and, more broadly, in the *Extended Evolutionary Synthesis* (EES) [200]. EES is not based solely on a gene-centric view of life, but instead takes the organism, its development (evo-devo), behavior, epigenetic inheritance, niche construction, and phenotypic plasticity more seriously than its predecessor, the Modern Synthesis of the mid-20th century [201,202].

However, it is unclear what role *agency* plays in *Major evolutionary transitions*, particularly the transition from unicellularity to multicellularity. Indeed, its role is still largely questionable, especially in multicellular organisms. For example, in a recent article, Newman et al. [192] discussed the connection between *agency* and organizational properties in multicellular organisms. The surprising revelation they came across is that this relationship between organization and agency is surprisingly less strict than that of individual cells in unicellular organisms. The main reasons for this are previously unrecognized morphogenesis of multicellular structures and the ability of development to amplify and distribute the functions of individual cells. These dynamics create new phenotypic capabilities that broaden the potential for agential behavior.

It is also evident that cybernetics and nonlinear dynamics offer a conceptually straightforward, though not mathematically or scientifically simple, perspective on purposeful behavior and the agents that embody, modify, and adapt those behaviors. As Vane-Wright and Corning [203] demonstrated in their overview of the *teleonomics* in the context of the life and work of Colin Pittendrigh, Raymond and Denis Noble [204] reveal an exciting truth: all living systems continuously exhibit creativity to maintain their integrity. To truly thrive, they must embrace and adapt to the ever-changing conditions of their surroundings.

This purposefulness, life’s most distinctive attribute, together with a shift away from the organism and the thermodynamics of adaptation in favour of selection, which Darwin denied, led evolutionary thought to become distanced from life itself [205,206]. Here, *teleological causality and purposeful dispositions* are not meant to reflect an outdated belief in teleological essence, such as the *élan vital*. Instead, as shown by García-Valdecasas and Deacon [207], this form of causality, reframed as *teleonomy*, can be demonstrated by a simple molecular process called autogenesis, in which two linked, complementary self-organizing processes give rise to higher-order relations expressed as constraints on molecular processes.

For better understanding, an example from the molecular physiology of the endoplasmic reticulum (ER) will help clarify *autogenesis*. The ER contains many enzymes involved in lipid synthesis. Moreover, as lipids—the most complex and enigmatic of the biological macromolecules—are manufactured in the ER, they are inserted into the organelle’s own membranes, partly because lipids are too hydrophobic to dissolve in the cytoplasm [208,209], (https://moviecultists.com/). Transmembrane proteins are also inserted into the membrane during synthesis, as they possess sufficient hydrophobic surfaces (https://www.nature.com). While transfer lipid proteins move lipids to mitochondria via non-vesicular transport, lipids and proteins are transported to the Golgi apparatus for further modification and packaging via vesicular transport [208]. Enzymes responsible for further modification and maturation of lipids in the Golgi apparatus are synthesized in the ER and then transported to the Golgi [210].

The reviewer of this paper proposes an interesting example with far-reaching consequences: the ER, in which vesicles packed with lipids and enzymes assemble, imposes the constraint that lipids with similar properties must be selected for assembly and synthesis. This might be a concrete example of autogenesis and molecular constraints in subcellular biology. However, I must add that there are a large number of these constraints in a single cell, allowing the synthesis of 10^9^ lipid molecules of different shapes and sizes just for the cell’s plasma membrane [209]. They must each have their own timing, act precisely on their specific molecular targets, and not interfere with other similar lipid synthesis pathways. More will be said later about the epistemological and causal roles of constraints in science and biology.

There are other examples of *goal-directedness* relevant to biology, which are likely triggered by emotional regulation of *goal-directed behavior*, such as emotional feeling, at least in humans. According to the action tendency prediction–feedback loops framework, this may increase the action tendency towards a goal or certain behavior [211]. For example, behavioural thermoregulation (body temperature homeostasis), which is associated with cognitive, physical, affective, and behavioural states, allows an animal or a human to actively seek shelter in the shade or cool down in water—an excellent example of goal-directedness. It involves different hierarchical brain structures, such as the prefrontal cortex (PFC) and hypothalamus. The prefrontal cortex, a uniquely human structure, regulates higher-order executive functions, including decision-making, social context adaptation, personality traits, and attention [212].

The hypothalamus contains the main thermoregulatory centre, considered a lower-level structure in the brain hierarchy [213]. The consequences of behavioural cooling, which is more energy-efficient than autonomic processes such as sweating and shivering, initiated by the PFC and hypothalamus, extend from the organ level down to cellular and molecular changes, providing another example of “top-down” causality. However, much work remains to clarify the connection between emotions and thermoregulation, which enhances survival in challenging or extreme conditions and serves as a good example of *goal-directed agential behavior* [214].

### 4.3. Haken’s Synergetics

*Agency*, *enactivism*, and later formulations of *synergetics* are closely interrelated. But what is *synergetics*? Physicist Hermann Haken is considered the father of synergetics, while theorist Juval Portugali has contributed to its further improvement and development. *Synergetics* is a theory that explains how synergistic interactions between components at lower levels give rise to emergent macroscopic collective behaviour. I have already provided a remark by Portugali that Haken, on multiple occasions, anticipated the “top-down” effects on lower-level components, which differentiates this theory of *self-organization* and complexity from others [78], p. 5; [215], p. 151:

“The slaving principle”. Once emerged, the order parameter enslaves (i.e., determines, describes, and prescribes) “the behaviour of the individual parts (like a puppeteer who lets the puppets dance).”

This led Portugali to conclude that Haken essentially realized that cognitive and brain systems, besides being complex, are characterized by “top-down” dynamics. These systems are active and, as such, actively participate as a kind of “inference machine” that initiates predictions about the environment in a top-down manner, then compares them to bottom–up information (embodied action-perception) [78,216]. In other words, living memory systems construct their dynamic spaces using information from the past, and their actions, supported by this information, are shaped by instincts, decisions, tendencies, or default ways of dealing with new situations or environmental challenges, based on solutions stored in memory [78]. To support this contention, Portugali [78] uses exploratory behaviour as an example: rats placed in an open-field arena exhibit exploratory behaviour in a novel environment until habituation sets in. This arena is also used to test (psycho)locomotor and anxiety behavior, with or without pharmacological modulation of neuronal circuits.

This type of behavior represents the behavioral phenotype and active “top-down” process that, in nature, leads to *niche construction*. More simply, as the author has some experience in behavioural neurobiology, I add that it enables the search for food, shelter, and mating partners. In biomedicine, exploratory behavior is used in the open field test, Y-maze test, and elevated plus maze test to study locomotor behavior, anxiety, working memory, and spatial recognition memory in ageing and neurodegenerative diseases (see [217,218]). These last two references also highlight the loss of information stored in memory in cases of ageing and Alzheimer’s disease, which affects systems involving personal memory. Another case of memory loss will be introduced at the end of this subsection. The earlier example of thermoregulation, introduced to illustrate *agentialism* and “goal-directedness,” can support a “top-down” hierarchical brain processing that influences lower-level brain structures and other organs down to cellular and molecular hierarchies.

According to Portugali [78], Haken’s unfinished project was to reframe *synergetics* to include the distinction between ordered *self-organized integration* (SOI) and ordered *self-organized disintegration* (SOD), or to expand *synergetics* to include memory systems. SOI holds that the interaction between unrelated parts of a system (i.e., a state of chaos) can produce an integrated system. Disintegration means that systems lose some of their characteristics or functionalities.

The crucial difference between the two is that SOI is a property of complex physical-material systems whose loss of properties, created through pattern formation, is random and that do not possess the memory on which synergetics 1.0 was developed (e.g., lasers). SOD characterises systems (living systems or hybrid engineered Hybrid Control Systems) with personal memory of individuals and collective-historical memory, using Synergetics 2.0. In other words, SOD in living memory systems happens with a sense of order. The *subemergence* of physical properties, on the other hand, is an example of the deactivation of properties.

For example, lasers, Bénard cells, and Prigogine’s dissipative structures are used to depict non-memory systems in *synergetics* 1.0. Haken and Portugali [216] have used the example they call “Synergetic Cities”, which, among other things, involves human (psychological) agents that possess memory. In this paradigm, the mutual nontrivial interactions among urban agents (such as physical, social, economic, and ecological components) give rise to an urban order parameter that “enslaves” (describes and prescribes) the behaviour and actions of the urban agents, and so on, in a circular causality. This is reminiscent of the theory of *autopoiesis* in biology. Of course, this model also distinguishes between many local structures and fast and slow processes in the urban system that give rise to a systemic whole controlled by this master control parameter.

This order or control parameter is a crucial part of *synergetics*. For example, if an organized network self-replicates, this parameter indicates that it is in the growth phase. In a network undergoing self-replication, the control parameter is undergoing growth. However, when this parameter exceeds a threshold, whether in decay or in rise, a phase transition occurs in the network. This is particularly interesting for the study of physiological networks and their emergence, subemergence, and redundancy, as I will discuss later in the section on emergence.

That is why SOI and SOD are introduced. Portugali [78], for instance, provides an excellent example of the tension between SOI and SOD. The first ancient urban centres in Egypt or Mesopotamia emerged by connecting and integrating settlements into a global urban network under the rule of a single centre (control parameter), which existed in a steady state for a relatively long period before disintegrating and receding towards nomadization. Here, I want to make some remarks that build on this argument. When considering the concept of SOD in humans and the rise and fall of civilizations, it is important to ask: Is the decline of civilization a result of collective memory loss in the context of SOD, or is it simply a random occurrence?

Civilizations and their accumulated knowledge often decline gradually, except when a sudden catastrophe triggers an immediate collapse. According to historians, the Roman Empire declined and fell over the course of centuries before finally collapsing. Of many kinds, then, the civilisation has lost the knowledge of how to make enduring Roman cement for construction. Thus, the loss of knowledge—such as the techniques for building pyramids—in scenarios of nomadization is a gradual and systematic process influenced by human decisions, actions, or the lack of appropriate actions within the urban system, which eventually breaks down into its fundamental components.

Another interesting example of synergism relates to our current environmental crisis. It concerns the study of synergetic control of carbon neutrality and network resilience through carbon metabolism in coastal regions of China [219]. The authors of this study set out to uncover the critical nodes and paths for synergetic control of carbon neutrality and to identify crucial components related to network resilience to provide a cleaner environment.

### 4.4. Rosenean Complexity, Miller’s Theory, Beer’s Model, and Beyond

Rosen is best known for his metabolic-repair (M, R) system. It formalizes fundamental autonomic features of life that are difficult to deduce from conventional biology [220,221,222,223]. However, to answer specific questions about particular organisms in research, it is necessary to use the (M, R) system—which addresses Rosenean or relational complexity—to identify a range of species-specific relational concepts. This approach aims to “recover the material systems through a process of realization of the formal system” [222], p. 1.

Annotation of (M, R) system is as follows: M stands for *metabolism*, a process applicable to all cells, while R stands for repair by using metabolic products to rebuild the machinery that supports metabolic processes, as well as to repair damaged cell components [220,221,222,223]. In short, the (M, R)-system causally captures the autonomous production of system components by other components and accounts for cellular homeostasis. At the same time, they are closed to *material causation* because of a net, overall, irreversible process that provides the thermodynamic driving force for metabolism [127].

Rosen’s groundbreaking work on biocomplexity is a vital effort to establish a theory that highlights the significance of *formal* and *final causes* in our scientific understanding of nature, mirroring Aristotle’s understanding of *physis* [223]. In Aristotle’s philosophy [224], p. 205:

“There is the unity of the efficient, formal, and final principle, as the ontological cause of the organism, which is called the ‘soul’ (psyche), while the material principle can be understood to represent its ‘body’ (soma).”

Biological organisms are dynamic and complex, maintaining stable equilibrium (homeostasis) and organization despite changes in their matter and form (through metabolism and metamorphosis) and challenges posed by changing environmental conditions [194]. Rosen was aware of the dynamic nature of the bidirectional relations between the organism and the environment. However, these relational and processual ontological ideas are much older and, in the twentieth century, were held by Alfred North Whitehead and the American naturalist or pragmatist Justus Buchler.

By focusing on the relational diversity and dynamism within and between complex life systems, rather than maintaining a static viewpoint (substance thinking), it becomes much easier to develop and implement nonlinear dynamical modeling strategies in the life sciences. Rosen was insightful on this issue, as nonlinear strategies are now the backbone of modeling complex phenomena such as weather patterns, pathogen spread, neurobiological processes, and more. Indeed, as Kineman has argued [223], Rosen’s system is designed to unify two theoretical aspects: ‘modeling relations’ and ‘category theory’ causal mappings. The former explores the intricate connections among “entities”, whereas the latter translates these relationships into formal mathematical structures.

Miller’s *living systems theory* (LST), extending Ludwig von Bertalanffy’s *general system theory* (GST), is an important framework that identifies multiple physical variables related to the *self-organization*, matter-energy exchange and flow, and the control of information in a hierarchy of eight levels: cells, organs, organisms, groups, organizations, communities, societies, and supranational systems, with 20 sublevels (subsystems) within each level [225,226,227,228]. This specifies cross-level formal identities in biological hierarchies and other contexts arranged by input–throughput–output processes [225,226,227,228].

Detailed lists of the functional elements of Miller’s system, classified under the following three processes—*Information Processes*, *Material-Energy Processes*, and *Processes that Occur in the System’s Output Stage*—apart from Miller’s extensive work, can be found in Elaine Parent’s (2020) seminar presentation and Vincent O’Rourke’s paper [229]. More specifically, at the cellular level, some of these elements in a bacterial cell are: ribosomes (producers), compartments such as the cytoplasm (distributor), and the cellular membrane (boundary), which acts as the interface for receiving and transmitting signals (information). In multicellular eukaryotic organisms, for example, the heart acts as a converter, transforming the energy stored in chemical bonds (such as glucose) into muscle contractions (motor) [228].

With later insights from Norris’s concept of *hyperstructures* and Holland’s *modularity*, it can be suggested that the subsystems of living hierarchies should be expanded to include functional modules and hyperstructures within cells. Cells display signs of differentiation into compartments, modules, hyperstructures, and condensates within the cytoplasm, nucleus, and organelles that process matter, energy, and information. These microorganizational aspects must be incorporated into any prospective computational model of the living cell.

Drawing on the work of cyberneticists such as Norbert Wiener, Ashby, and others in the late 1950s, Stafford Beer proposed the cybernetic *viable system model* (VSM), a system “capable of independent existence” to account for a holistic observation of collective behaviours in societies [230,231,232]. Drawing on mathematics, psychology, biology, neurophysiology, communication theory, anthropology, and philosophy, his model identifies an organization’s critical variables and, based on this set, determines the directions for placing effective homeostats to monitor these variables and maintain equilibrium within a natural or socio-technical system [231,233]. More specifically, VSM draws on the structure of the human nervous system to model socio-technical or natural systems as neural networks, with each node as an autonomous, viable system, and all nodes together forming a purposeful and cohesive whole [232].

Beer imagined his model as a way to determine or design, in the case of a socio-technical system, the most appropriate organization to ensure the system’s viability, adaptability, and identity, or “to mimic the evolutionary strategies to manage complexity and achieve long-term viability” [234], p. 705. Although Beer considered that viability allowed the evolution of the system, he failed to explain in detail how viability and evolution are linked [235]. The value of the VSM model lies in its applicability to any “viable system, from the individual to the small group to the organization and so on. The only criterion is that each level has the potential to support itself as an independent entity” [233], p. 575. In other words, entities that are embedded within other entities (Beer’s embedment recursion) perform in a manner appropriate to their scale, with the appropriate respect for their environment and larger entities (ibid.).

The basic mutually interactive components of the VSM model (partially modified), according to Espinosa et al. [232], p. 2; (https://youtu.be/gPnWVg7CSIg, accessed on 15 March 2026), are:

1. Operational Units (O): The elements of the biological system or organization directly responsible for implementing its purpose. Operations carry out all the basic work of the system.
2. Environment (E): The niche to which the organism or organization is structurally coupled and with which it co-evolves. External conditions the system as a whole operates within the viable system.
3. Meta-System (M): The managerial and technical support required to coordinate the operational units and provide them with the resources, technology, and knowledge necessary to perform their tasks. The meta system ensures cooperation, integration, and forward planning across the entire system.

The first two points appear to model genetic-environment interactions (G × E), the process by which genetic expression is altered by the environment. In a biological context, the third point concerns Systems 4 (Intelligence/Environment) and 5 (Policy/Identity), where, for example, higher brain functions affect long-term, purposeful decisions, data collection, and planning. The list of VSM systems modeled on the human organism, according to Google AI overview and The Viable System Model|A short introduction to Stafford Beer’s Viable System Model (VSM) and its potential use today (https://youtu.be/gPnWVg7CSIg), is:

System 1 (Operations): The muscles, internal organs, and autonomous bodily functions that engage directly with the environment.

System 2 (Coordination): The sympathetic nervous system, which harmonizes the activity of muscles and organs to ensure stability.

System 3 (Control/Management): The autonomic nervous system/medulla (base brain), which manages and optimizes the internal interactions of the muscles and organs.

System 4 (Intelligence/Environment): The conscious nervous system/diencephalon (middle brain), which senses the outside world, collects data, and plans for the future.

System 5 (Policy/Identity): The cerebral cortex (higher brain), responsible for identity, long-term decisions, and overall purpose.

Regarding the modularity of CAS and Norris hyperstructures within the cell (discussed in the next section), it is hard to determine whether these functional agglomerations constitute viable entities. Beer considers the cell as a minimally viable entity. Therefore, although VSM draws inspiration from biology, it cannot fully account for the subcellular domain. It needs these other frameworks to help address how this domain self-organized.

Rob Dekkers [236] has recently proposed a cybernetic steady-state model that connects LST and VSM, allowing systems-theoretical referencing to enhance self-criticality in adaptive structures and processes beyond biology. This model depicts regulatory and control processes between agents in networks within broader organizational and engineering systems, and also provides an explanatory concept for self-criticality in complex adaptive systems. It shows us how internal variables in systems remain constant despite external changes through negative feedback loops, which act as stabilizing control mechanisms. Steady-state models (SSMs) in fact utilize biological homeostasis, which Dekkers tried to depict in other (business) process modelling approaches for operations management. SSM has the potential to extend LST and VSM, which are grounded in biological *self-organization*, to sociotechnical systems. In other words, Dekkers has shown us how biology and the philosophical frameworks built upon it can help develop complexity theory and organizational science.

Metascientifically, LST, von Bertalanffy’s General Systems Theory, VSM, SSM, SOC, and CAS share many similarities and some differences. While Miller’s theory and GST focus more on the “wholeness” of hierarchies and how multicomponent interactions produce these levels, SOC, CAS, VSM, and SSM, in the spirit of general cybernetics and chaos theory, focus more on explaining how these structures and levels emerge through non-linear interactions and positive and negative feedback loops [237]. VST, unlike these other theories, has a much stronger foothold in business theory, management, and politics. For instance, Leonard [238] uses the metaphor of biological symbiosis to help troubled countries and societies become economically and socially viable through VSM.

## 5. Emergence in Biology

The concept of emergence is both quintessential and challenging to study, model, and explain. Historically, it is often associated with the British philosopher G.H. Lewes, who referred to J.S. Mill’s idea of “heteropathic” or “non-additive effects” in nature [239], p. 371. The concept was further developed in the 1920s by the British emergentists C.D. Broad and Samuel Alexander [240]. It proposes that higher-order phenomena, including life and consciousness, depend on, yet are autonomous from, the underlying physical reality [241]. This is the most basic and broadest interpretation of its moderate form, which has recently seen a resurgence in complexity science.

The distinction between *weak* (epistemological) *emergence* (WE) and *strong* (ontological) *emergence* (SE) has already reached the status of a classical one in any serious discussion of complex systems. According to philosopher David Chalmers [242], p. 244, SE occurs when a higher-level phenomenon builds on the lower-level domain, but “truths concerning that phenomenon are not deducible even in principle from truths in the low-level domain”.

In contrast, WE proposes that “the high-level phenomenon arises from the low-level domain, but truths concerning that phenomenon are unexpected given the principles governing the low-level domain” (ibid,). In other words, WE explain how new properties and behaviors arise that cannot be predicted or explained from knowledge of the individual parts. SE, in particular, is what philosophers and scientists have in mind when they engage with the true ‘complex systems’ (see, for example, [196,243,244,245]).

Making sense of both WE and SE in the dynamics of complex systems presents a significant challenge for academia, sparking lively debates in science and philosophy. In the field of artificial intelligence, philosopher Marc Bedau [246,247] developed and discussed WE as a strong form of epistemological emergence, independent of the psychological and logical limitations of the human mind. According to Thorén and Gerlee [248], who provide a critical assessment of Bedau’s work, Bedau argued for WE based on a simulation requirement and an appeal to explanatory incompressibility. The former concerns simulation governance, that is, creating the conditions to ensure and enhance the reliability of predictions from numerical simulations through verification procedures, measurement or collection of physical properties, ranking of mathematical models, and more [249].

The latter, according to Bedau [247], p. 443, accounts for the idea that “systems’ macro properties can be explained by their micro properties but only in an especially ‘complicated way’.” The interconnectedness of Bedau’s two forms of WE allows us to approach “complex, macro-pattern in the mind-independent objective micro-causal structure that exists in nature” (ibid.). In metascientific contexts, a key issue in the philosophy of complexity is how to develop a theory of “non-epistemic emergence” that is compatible with mechanistic explanation but incompatible with reductionism [250], p. 277.

John Conway’s *Game of Life* (GoL), a primer on “cellular automata” first introduced to the public in 1970 to demonstrate how simple rules can generate complex and adaptive behaviours, is perhaps an example of what we discussed and used by Marc Bedau [244,247,251,252]. In this *Game of Life*, the time evolution of certain simple Life configurations suggests that some configurations remain unchanged forever (so-called “still lifes”), some oscillate indefinitely (so-called “blinkers”). In contrast, others continue to change and grow indefinitely (increasing in the number of living cells) [247]. The main point is that all these configurations are determined by the system’s microdynamics, the simple birth–death rule, and the world’s initial state configuration. Bedau [247], p. 381 is clear about earlier weak emergence in GoL: “It follows that a structural macrostate in Life will be weakly emergent if deriving its behavior requires simulation”.

*Strong emergence*, which according to Bedau [247] can be accused of having relationship with mysterious irreducible downward causation, involves (1) the “occurrence of qualitative novelty” [253], p. 14, (2) the “degree of reality or autonomy over and above the set of its base elements” [254,255], (3) the “dependence relation between the source of emergence (the emergence base) and the result of the emergence (the emergent phenomena)” [254,256], p. 214, and (4) *holism*, the belief that the whole is more than the sum of its parts [256]. Here, emergent relata may include emergent processes, activities, interactions, emergent entities or systems, and emergent properties and relations [256], p. 219.

Drawing on Humphrey [257], Glennan [256], p. 218 introduces the distinction between ‘producing’ and ‘underlying’ emergence to further refine the taxonomy beyond the established WE and SE. Compared to these, it provides, on one hand, deeper insight into the reasoning behind the evolved space-time-related multiple interactions of low-level molecular components, and, on the other hand, into the synchronous relationship between specific low-level mechanisms and the emergent properties on which these properties supervene in every possible case at the moment of observation and measurement. It describes a situation in which we identify an *emergence* base (*diachronic emergence*; *producing emergence*) as the set of “startup conditions which, via an etiological mechanism, produce the emergent phenomenon” (ibid).

Glennan maintains that “the emergence base is temporally prior to and distinct from the emergent phenomenon, and the etiological mechanism is the causal process by which the emergent phenomenon arises” (ibid.). In *synchronic emergence* (underlying emergence), by contrast, the emergent phenomenon depends upon an underlying mechanism, which coexists with the phenomenon in space and time’ (ibid.). This distinction, and Glennan’s [256] work in general, not only deepens our understanding of *emergence* but also demonstrates how to address the tension between emergentism and mechanistic philosophy to improve their less-than-ideal connection within what he calls the *Mechanistic Emergence* framework. In this way, mathematical modeling of mechanisms may gain new impetus, especially in understanding how to translate the quantitative nature of mechanisms into emergent qualitative novelties. The down-to-earth example of “producing” and “underlying” emergence is, according to ChatGPT:

“A hurricane has an ‘emergence base’ comprising warm seawater, Coriolis forces, etc. that exist before and are distinct from the hurricane that emerges, whereas the etiological mechanism or organized causal process by which the base produces the emergent phenomenon includes heat transfer, convection currents, rotation, etc.”

I now wish to highlight a crucial distinction between the two types of emergence, which is essential for understanding that there are, essentially, two parallel approaches to emergence: *Being emergence* and *pattern emergence* (pattern formation). This distinction, presented by Jason Winning and William Bechtel in the Routledge Handbook of Emergence [258], is crucial for avoiding confusion between philosophical and scientific perspectives on the subject. It separates the speculative from the practical (scientific) aspects of *emergence*. As crucial to this paper, it outlines two interconnected yet distinct approaches to studying emergence pursued by philosophers and scientists.

The first approach is philosophically affiliated and lacks consensus. It is based on the longstanding debate about the gradation of Being, prompted by Aristotle, Plato, Descartes, and later philosophers, with significant implications for philosophical monism and pluralism. The second approach is *pattern emergence*, which is the primary focus of scientists and modelers. The main difference is that, while scientists seek to capture the real-life manifestations of emergent patterns in physicochemical and biological systems to explain transitions from the micro to the macro level, philosophers have debated the ontological and epistemological consequences of emergentism, more explicitly after the British emergentist movement at the beginning of the twentieth century.

Nevertheless, these two types of emergence inform one another in the joint search for a comprehensive understanding of the macroscopic reality comprising both living and non-living things. At the end, one reality, one knowledge drives both philosophy and science, and it is a fundamental assumption of the complementarity between these two domains. This is effectively demonstrated by ongoing efforts to connect emergence with other key concepts, such as nonlinearity and self-organization, which are essential for understanding complex biological systems.

One notable example of *Being emergence* is Plato’s theory of Ideas. He suggests that there are intelligible, immutable, and timeless abstract entities known as Forms or Ideas, while the physical world consists of mutable beings that merely reflect these Forms (see [259]). Understanding a “gradation of beings,” or how these Ideas and the concrete things that reflect them constitute reality, is one of the most challenging problems in philosophy.

By contrast, Wegner [239], p. 369 recently presented an interesting example of *pattern emergence*. He argues that a network of coupled fluxes of matter, free energy, and entropy in living organisms, which he refers to as “Metafluxes,” can be described and axiomatized by the thermodynamics of irreversible processes. His model diverges from metaphysics and the way *emergence* is considered there simply by establishing a clear connection between metafluxes and both WE and SE, treating them as non-exclusive concepts. In doing so, it directly links emergence to fundamental thermodynamic concepts such as matter, energy, and entropy. In addition to reconciling SE and WE, his account provides a rationale for *pattern emergence*. Other examples of this type of *emergence* include temperature, magnetism, herd immunity in social networks, and so on [260].

Consciousness, with its so-called “hard problem of consciousness”—the explanatory gap between the brain and *phenomenal consciousness*—serves as a unique example of *emergence* because it represents the battlefield where SE, WE, *Being emergence*, and *pattern emergence* confront and compete. The “hard problem of consciousness’ concerns how the complex, non-trivial interactions among billions of neurons (matter) give rise to *phenomenal consciousness* and self-awareness over time [261]. This issue has sparked intense metaphysical debates, from ancient Greek philosophers to the present, leading to conflicts between *physicalism*, *dualism*, and *epiphenomenalism* [262,263]. Conceived in this way, consciousness has serious philosophical and ontological implications: the type of *emergence* involved here is *Being emergence*. It is worth noting that *physicalism* holds that consciousness is a product of underlying biology and, ultimately, physics.

In contrast, dualism, following Descartes, draws a sharp distinction between the material world (res extensa) and the realm of the soul or consciousness (res cogitans). *Epiphenomenalism* recognizes that consciousness arises from brain activity but denies that mental events can cause changes in the material, physical world. Not all philosophers agree that consciousness is strongly emergent. In a recent article, Eli Haitov [264], p. 1 proposes:

“Since it is allegedly a brute fact that emergent properties arise in certain complex systems, they should emerge in anything. Since they do not emerge in everything, they also do not emerge only in certain complex systems.”

Therefore, he is clear that WE is what both scientists and philosophers should subscribe to when dealing with consciousness. Cognitive scientists and neurobiologists, however, focus on understanding how interacting, self-organized neural networks generate and sustain conscious experience. These neuronal ensembles, circuits, and networks are functionally connected under the umbrella term “connectome” [265]. In the mammalian brain, a balance between cooperation and competition among distributed circuits maintains functional connectivity [265]. *Cooperation* entails alignment between components, e.g., neuronal states, while competition refers to negatively correlated goals of agents, such as neurons in a circuit. *Cooperation* and *synergism* produce new states and emergent properties, while competition and other stabilizing processes enable the stabilization and optimization of system dynamics.

In ecology, particularly microbial ecology, *cooperation* among groups of microorganisms is an emergent property influenced by energy supply and residence time. Gralka et al. [266], p. R1179 describe two scenarios in which *cooperation* and *competition* interact. The first scenario involves a high resource supply, often accompanied by high dilution rates (resulting in low residence times) in continuous or semi-continuous culture conditions to prevent the accumulation of biomass waste products. Under these conditions, primary consumers of the supplied resources excrete a diverse range of primary metabolites that support a variety of secondary consumers. The survival of these secondary consumers depends on the extent of competition for these metabolites.

In contrast, the second scenario arises when resource supply is low. Here, dilution rates must also be low (resulting in high residence times) for slower-growing organisms to persist. In these conditions, resource limitation affects all species, and groups of organisms that can effectively complement each other through their metabolic excretions are more likely to thrive. This cooperation enables informational synergy among heterogeneous groups of microorganisms, or more broadly, among different cooperating agents [267]. Pattern emergence best captures this example.

In a seminal work on *reductionism*, *holism*, and *emergence*, Massimo Pigliucci [268], pp. 264–265 introduces two examples of *pattern emergence* that suggest important ontological and epistemological implications. The first example is based on the mathematical paper by Romero and Zertuche [269] and involves NK networks, or Kauffman-type networks (introduced in 1969). These networks are *cellular automata* used to explore the properties of genetic networks, which are characterized by N elements, each with K input connections and one output. As Pigliucci interprets it, robustness—something we will also discuss later—emerges from the statistical properties of a genotype-phenotype modelled as an NK Kauffman-type network. Interestingly, emergence in this context is described as the “appearance of a biological property (robustness) resulting from specific non-linear interactions among lower-level entities (the genes in the network)” [268], p. 264.

The second important example provided by Pigliucci [268], p. 265 involves genetic-environmental interaction (G×E, or G-by-E) and the reaction norm diagram. This diagram allows us to disentangle and appreciate the average effect of the environment on a given trait, which can be quantified by Environmental (E) and Genetic (G) variances. The G-by-E interaction variance arises from statistically *nonadditive effects* that cannot merely be summed from genetic and environmental influences. Thus, “A population with a significant G-by-E variance, therefore, exhibits a quantifiable ‘emergent’ (at the statistical, population-level) property’ (ibid.).

The ontological implication of Pigliucci’s examples is that at least some biological properties are ontologically emergent, while the epistemological implication is that we need mathematical, statistical, and computational reasoning (in the case of NK) to explain the specificities and processes that underlie their occurrence.

In their work on combined instances of WE, SE, and *pattern emergence*, Feinberg and Mallat [270] use complex systems theory to examine the emergent features of life and, subsequently, complex brains. They describe three progressive levels: Level 1 (Life), Level 2 (Nervous Systems), and Level 3 (Special Neurobiological Features). Each level marks an increase in biological and neurobiological complexity, ultimately leading to the emergence of phenomenal consciousness within physical systems. Along this trajectory, they show that consciousness is an emergent property, albeit one of extreme complexity. The relationship can be summarized as: Life + Special Neurobiological Features → Phenomenal Consciousness.

Perhaps the reconciliation between WE, SE, and *pattern emergence* in biology may require a more conceptual approach after all. Luisa Damiano’s [271] dual solution to *emergence* in the biological realm—specifically, the *theoretical problem* (models of emergent properties) and the *epistemological problem* (ensuring the scientific value of these model descriptions)—can help reconcile the emergentist view of life with an emergentist understanding of science. More concretely, scientists must identify brain patterns that contribute to consciousness and use all available interdisciplinary resources to study and model mental states.

## 6. Emergence via Coherence and Redundancy

### 6.1. Connecting Coherence and Emergence

The main idea behind relatively recent nonlinearity-based frameworks, such as coherence and bifurcation, is to explain and formalize qualitative changes in the behaviour of dynamical systems, particularly where abrupt transitions, such as those in the evolution of life, may occur in evolving systems (e.g., qualitative behaviours of biochemical reactions reflecting synthesis and degradation of molecules) [272,273,274]. Understanding the concept of *coherence*, which draws on chaotic behaviour, nonlinear dynamics, solitons, and bifurcation theory, is vital. *Coherence*, coherent nonlinear dynamics, and coherent structures are crucial for understanding dissipative, externally driven nonlinear systems [275,276]. More broadly, coherence is a macroscopic property or collective state that acts as an efficient mechanism for biological self-organization [277]. In this context, it results from thermodynamic openness (the expulsion of entropy into the external environment) and refers to energy transfer and information processing within molecules [277]. In this paper, we will discuss biological examples of *coherence* that illustrate its connections to *emergence*, without delving into mathematical or computational aspects of the challenges it raises.

The study of coherence in biology explores two important avenues. The first, which I call “supramolecular coherence”, concerns the functional organization of molecules within cellular compartments, including modules, hyperstructures, condensates, and related structures. The second group examines the effects of quantum-biological coherence on critical biomolecules, including those involved in plant photosynthesis (photosystem I and II), animal and plant magnetoreception (cryptochrome), neuronal microtubule, enzyme catalysis, immunology, and evolution [278,279,280,281].

The majority of physiological, pathological, and ecological processes, such as metabolic cycles, sleep–wake patterns, endocrine physiological rhythms, cancer, cognition, and collective ecological dynamics within large groups of organisms, can best be explained in terms of *coherence* [277,282,283]. At present, the first type of *emergence* is the most likely candidate to explain many important features of life’s inner workings, but recent advances in quantum biology have made the second type of emergence harder to rule out. In other words, I will not claim that *quantum coherence*, *superposition*, and *entanglement* do not play an important role in emergent biological processes. However, I will remain within the field of molecular cell biology to address *coherence* without delving deep into quantum physics or quantum biology.

The first type of *coherence* holds significant promise for emergence. However, I have concerns about the second type, as many remain skeptical of the nontrivial transmission (percolation) of quantum effects through biological hierarchies [284]. The main suspect for the lack of quantum effects on the macroscopic scale seems to be *quantum decoherence* in a noisy environment, such as a cell, and the strong coupling between the organism and its environment. Despite this apparent skepticism about quantum phenomena in biological systems, recent years have ushered in an exciting era of quantum biology, as quantum studies are now experimentally capable of measuring degrees of quantum entanglement and coherence [285,286].

McFadden and Al-Khalili [287] proposed that the accelerated mutation rate and the production of mutated states in microorganisms may be driven by *decoherence* through quantum tunnelling of protons within DNA hydrogen bonds. The genome remains stable because *quantum coherence*, or *quantum superposition*, can be maintained for biological timescales until *decoherence* occurs. This *decoherence* alters the quantum superposition of the genome and entangles it with its environment, potentially leading to mutagenesis. However, understanding quantum processes in macroscopic biological systems is fraught with experimental and theoretical uncertainties and warrants further investigation.

Let us first discuss the first type of *coherence*. In cellular biology and microbiology, *coherence* enables meaningful (i.e., selectable) phenotypic diversity—reflected in the diversity of growth rates—within a cell population, where cells avoid attempting to grow and sporulate simultaneously [288]. It requires that each phenotype be consistent with the set of genes expressed [289].

*Hyperstructures*, a concept introduced by Norris et al. [290,291], p. 313 and further developed by Norris et al. [292], propose that “hyperstructures constitute a level of organization intermediate between macromolecules and cells” and that, with different turnover characteristics, they can explain how this coherence is achieved. As Gangwe Nana et al. [288] argue, coherent diversity in bacterial cells may be explained by the hypothesis that one parental DNA strand is physically associated with proteins appropriate for a survival strategy. In contrast, the other strand is associated with proteins appropriate for a growth strategy. This, together with the activity levels of these *hyperstructures*, represents an effective strategy for controlling the cell cycle [288,289].

Norris’s concepts of *hyperstructures*, in some sense, anticipated another conceptual breakthrough in cell biology in 2017, when Banani et al. [293] introduced the concept of biomolecular *condensates*. What is condensation in a cell? Simply stated, it is the universal process in nature by which molecular species segregate into dense and dilute phases (e.g., morning dew) [294]. In biology, *condensates* are subcellular micron- or submicron-scale dynamic structures in which functionally related proteins and nucleic acids assemble through liquid–liquid phase separation, allowing them to form on a larger scale without a membrane [294]. Structural and functional modifications of these *condensates* may play a crucial role in the aging process and neurodegeneration [295]. Historically, these structures have been referred to by various names, including membraneless organelles (MLOs) and granules [294].

Furthermore, Norris has developed the concept of *competitive coherence*, which is particularly interesting from the perspective of *emergence* and complexity. In *competitive coherence*, *emergence* should be understood as the formation of a new state, that is, the production of a subset of elements that are active together. Norris et al. [291], pp. 325–326 provide thought experiments to test how competitive coherence operates in the environment. Firstly, “environment acts via the coherence process to lend importance to one out of many sites”. Consequently, selection favours this site and the molecule that binds to it. They then ask: What if a protein with this binding site binds to a phospholipid to form a domain where their activities complement each other?

There may be selection among other complementary proteins for this site, which, theoretically, provide a range of “types of connectivity to determine membership of an Active set and this Active set would take on the physical form of a proteolipid domain responsible for a particular function” (ibid.). This led the authors to conclude that the terms of a new criterion for membership of the Active set provide a more practical understanding of emergence in cellular biology. In this context, they demonstrated a direct connection between emergence and competitive coherence. The significance of their work lies in recognizing clear relationships between *coherence* and *emergence*, which can help researchers understand the development of cellular organization and, consequently, multicellular biological systems.

### 6.2. Emergence, Redundancy, and Subemergence

I will begin this subsection with an example from genetics. It concerns the quantification of biological traits, which can more concretely account for *emergence*, *redundancy*, and *subemergence*. As genetics teaches us, multigenic complex biological traits (phenotypes) depend on emergent interactions among a proper set of proteins. The emergent interactions of proteins shape complex, multigenic biological traits, which are the main functional units at the molecular scale. Wegner and Hao [296] and Hao et al. [297], p. 841 have recently developed two algorithms for quantifying WE and SE. The WE algorithm is based on the premise of pairwise reciprocal interactions between proteins, in which each protein modifies its contribution to a complex trait in turn. The second algorithm assumes the formation of a new, complex trait by a set of n ‘constitutive’ proteins at concentrations exceeding individual threshold values (strong emergence).

The main assumptions of the algorithm for quantifying SE are protein redundancy with respect to a complex trait (full redundancy) and, above all, irreducibility, which holds that if one constitutive protein is missing or its concentration drops below a threshold, the trait is lost [297]. Protein *redundancy*, together with genetic *redundancy*, is fundamental for living organisms to cope with the harmful impact of the environment. It is fundamental to all organisms’ ability to cope with environmental stress and harmful mutations, providing resilience to living systems in terms of their structure and function [298].

Furthermore, this phenomenon is built on the assumption that in living organisms, there are proteins and genes whose functions are similar, overlapping, or even identical. In the case of a mutation, if one protein is defective, the other can take over its function [298]. The point is that, in the case of a multigenic trait, we cannot reduce the explanation of the phenotype to a single gene or protein, or to a group of them, without considering the whole picture determined by emergent interactions. *Redundancy* and a similar process, reciprocal-based robustness, play a critical role in cardiac and brain electrophysiology. Noble [299], pp. 2–3 has recently argued that cardiac pacemaker activity is formed from multiple interlocking physiological networks, any one of which can generate rhythm and automatically replace the others:

“In such interlocking control systems, the association scores for individual components are necessarily low, even though causation, measured by the electric current carried by the relevant ion channels, is large. This kind of reciprocally based robustness is widespread in living organisms, which explains why most association scores in genome-wide association studies are low, or even zero.”

More specifically, pacemaker activity depends on the functional hierarchy of pacemaker clusters in the sinoatrial node [300]. Noble’s ideas and long-term research have significant implications for genetic studies and offer a new antireductionist perspective on why understanding molecular biology is essential but not sufficient for grasping both healthy and diseased phenotypes.

The concept of regulation is central to understanding processes and properties such as *stability*, *robustness*, and long-term persistence, particularly in biology. Bich et al. [301] and Bich and Bechtel [146] proposed that, in biological systems, a specific subsystem responds to and manages environmental perturbations to alter the constitutive regime of a system without being specified by it (e.g., negative feedback systems) (see also [302]). Pinto Leite et al. [302] investigated whether such regulatory systems exist in ecology and concluded that they do not, possibly because ecological systems extend beyond the limits of dynamic stability and *feedback mechanisms*. All these principles are summarized in one sentence by Rahman [303], p. 3:

“The behaviour of living systems, from single cells to complex organisms, is governed by the integration of internal structure, energetic readiness, and environmental context”.

The process of losing emergent properties in the system is called *subemergence*. Elder-Vass and Zahle describe complex systems as comprising various entities that both combine with and dissolve into other entities. Bunge [253] notes that emergence results in the creation of new entities, while submergence leads to their dissolution, characterized by the loss of one or more emergent properties within the system. Recently, *subemergence* has attracted significant attention in the scientific community. Gianfranco Minati [304] from the Italian Systems Society discussed experimental approaches to deactivate emergence (de-emergence) when interventions in complex systems are necessary to mitigate their destructive effects, such as tornadoes arising from Rayleigh-Bénard convection.

In dynamic biological systems, many processes occur or are regulated through *emergence* and *subemergence* across various scales of biological organization. For example, their interplay can be observed in the cell. To maintain a healthy state or protect its proteome (the complete set of cellular proteins) from harsh environments, a cell must ensure proteome quality [305]. This is achieved through *proteostasis*, which includes protein synthesis and degradation, all of which are monitored by a network of guardian proteins to maintain homeostasis [305].

These regulatory genes and proteins protect the cell’s identity, which in turn supports the holistic identity of the entire organism. It is worth noting that protein degradation may or may not influence emergent properties at higher levels, due to the *redundancy* and *resilience* discussed above. However, not all traits are multigenic or result from protein interactions; for example, a mutation in the SMN1 gene causes *spinal muscular atrophy*. Here, *subemergence* affects specific emergent properties or traits necessary to maintain the integrity of motor neurons. In this context, protein loss within a cell may or may not illustrate the concept of *subemergence*, depending on the physiological and genetic context. The process of loss of properties is more evident in dying necrotic cells or in cases of programmed cell death (apoptosis).

*Subemergence* can also be illustrated by the idea of system collapse or cascading effects, which is of interest to biologists. As discussed by Portugali [78] and Buldyrev et al. [306], a Havlin research group, complex systems are also characterized by the proliferation of networks and interdependencies between them. If one network in the system fails, it often triggers the failure of another. For example, a power grid failure leads to an internet outage. The only unknown in the equation is how resilient the system is at compensating for network failures. As I have shown, some networks and elements of complex biological systems are more resilient than others. Evolution has enabled life to develop a pretty decent compensatory mechanism to confront the sudden failure of cell molecular machinery.

Notably, *cascade multiplicative dynamics*, in which the output of one structure or subsystem serves as the input to another in series, are important concepts in complexity-oriented biology. Research has shown that cascading dynamics in all their forms may be important for preserving robust multifractal scaling and for serving as a mechanism for constraining lower hierarchies, which represents a fundamental organising principle in biological systems. This includes processes such as cardiovascular dynamics, cerebral blood flow, neural activity, and brain–body interactions, respiratory behaviour, gait, and posture [307,308]. According to Mangalam et al. [308], p. 1, it manifests:

“As structured variability that exhibits scale-invariant structure across multiple temporal and amplitude scales to capture the complex interplay of regulatory mechanisms spanning fast, fine-scale adjustments and slower, larger-scale modulations—a hallmark of biological systems that must simultaneously maintain homeostatic precision and respond adaptively to unpredictable challenges.”

The phenomena of *coherence* and *redundancy* appear to be promising strategies for explaining how self-organized processes lead to strong emergence while remaining consistent with the Causal Closure of Physics (CCP) principle. CCP—the idea that all physical events must result solely from physical, not mental, causes, or, in a more epistemic sense, that every physical event has a physical explanation [309,310]—is not contradicted by the system. In other words, these, among other processes and phenomena, illustrate what Heylighen [311] describes as the reason why *emergence* and *self-organization* are conceptually simple, common, and natural. Emergence and subemergence seem to have cognate terms in systems science–SOI and SOD—in Hermann Haken’s synergetics, which are discussed in the subsection on synergetics.

In Haken’s and Portugali’s synergetics, physical systems without memory are prone to this form of property loss. However, SOD characterizes systems with memory, such as living organisms. I showed that a cell loses some of its properties in a self-regulated manner. This is absolutely true, for example, in the case of *apoptosis* (programmed cell death). However, the question is whether this is true for sudden, unprogrammed cell death, such as *necrosis*. By scratching at the surface, *necrosis* is also, to some degree, an ordered process, unlike the standard model, which denies its regulated nature. Recent research has reported that it is an active process regulated by cells destined for death, and that some of its mechanisms are connected with those of *apoptosis* and *autophagy* [312]. Ageing, neurodegeneration, ischaemia, and other forms of loss of biological properties involve up- and down-regulated processes.

## 7. Holism and Holistic Biology

### 7.1. Unlocking Holism Through Boundaries of Complexity

Now, let us focus on *holism* as the fourth listed characteristic of *emergence*. Most broadly, the claim that the “whole is greater than the sum of its parts”, first proposed explicitly by Aristotle and later advanced by Jan Smuts in 1926, is a significant philosophical principle central to the conceptual development of the term ‘ontological holism’ and its epistemological counterpart, termed “epistemological holism.” *Holism* or the concept of “wholeness”, fundamental to complex systems, is traditionally approached from two main perspectives: epistemological and ontological. *Ontological holism* holds that [313], p. 110:

“Holistic systems are such that their constituent parts have some of the properties that are characteristic of these things only if they are organized in such a way that they constitute a whole of the kind in question.”

In its broadest sense, the entire universe can be seen as a single, holistic system or monist entity, consistent with Neoplatonic concepts of “One.” *Epistemological holism* (confirmatory holism), which has existed for some time (at least from Quine onwards), maintains that a single model, theory, or hypothesis cannot be tested in isolation; instead, it depends on supporting auxiliary theories, with both primary and auxiliary theories tested together [313], p. 1. This form of holism will not be considered in this paper.

*Reductionism* is a contrasting concept to holism. It asserts that the “whole” is simply the sum of its individual parts, a view known as *ontological reductionism*. Consequently, the best way to explain the structure and function of the whole is by examining the interactions of its constituent parts (epistemological reductionism), while the most effective methods and approaches to understanding the whole focus on lower-level features and behaviors (methodological reductionism) (see [64]).

Laszlo and Krippner beautifully highlight the essence of *holism* in complex systems theory [314], p. 57:

“Structurally, a system is a divisible whole, but functionally it is an indivisible unity with emergent properties.”

For example, the brain, in both animals and humans, is the most complex organ in the body, largely due to its remarkable ability to store and process information. This complexity arises from a structured array of functional elements, including essential components such as the pons, thalamus, striatum, cerebellum, cerebrum, and hippocampus, which work together to enable the extraordinary capabilities of the mind. Still, its behaviour, function, and other properties “are more than the sum of the system parts at any particular level or across levels” [315], p. 204, or more than the sum of these brain structures.

Ludwig von Bertalanffy is also clear-cut about *holism* with a hint of methodological instructions for practical (scientific) interactions with complexity [316], p. 30:

“It is necessary to study not only parts and processes in isolation, but also to solve the decisive problems found in the organization and order unifying them, resulting from the dynamic interaction of parts, and making the behavior of parts different when studied in isolation or within the whole.”

Norbert Wiener, a founder of cybernetics, is also associated with *holism* by highlighting the ordered interconnectedness, synergy, and controlled processes among components that produce organized systems, such as living organisms or human-made cybernetic systems [317,318]. Moreover, Lorenz’s Chaos theory promotes a holistic understanding of complex systems by highlighting that the equations governing interactions within a system’s parts cannot always fully explain the system’s overall behaviour [319,320].

The understanding of the boundaries and meaning of a “holistic system” or “whole” depends on the criteria used to define it, which is directly reflected in what we consider complex systems. An essential yet often overlooked connection between holism and complexity lies in efforts to define the “boundaries of complexity”. Defining the boundaries of complexity expands our understanding of both concepts and their interconnectedness, ultimately fostering a greater appreciation of complex systems. Taken together, the steps we take towards charting the boundaries of a “holistic system” and the “boundaries of complexity” may have their roots in both our scientific methodology and practical actions, as well as in the metaphorically perceived evolution of humanity.

The former is articulated by Chu [321], p. 229, who states that “the modeller always needs to draw artificial boundaries around phenomena to generate feasible models.” Here, an intuitive notion of ‘radical openness’, which suggests that no systems exist naturally, is essential for establishing boundaries around the system. Instead, systems are formed through the observer’s active role in perceiving and interpreting reality. This reflects the “coupling between observer and observed,” a key idea in second-order cybernetics that shapes our decisions about what to consider a complex system (see [62,318]).

Chu’s other variable is “contextuality,” which appears to be “closely connected to the requirement to simplify models and to leave out most aspects” [321]. When these two—“radical openness” and “contextuality”—cannot be contained, that is, when it is not clear where the boundaries of the system are or which abstractions are correct, complexity arises.

The evolutionary explanation offers an even more intriguing perspective. At the core of the Darwinian evolutionary worldview of human cognition, or one of its paradoxes, lies the intriguing fact that humans evolved to respond to complexity in two simultaneous ways: to appreciate complex patterns positively and to distrust them. The evolution of self-awareness in early hominids, across many hominoid species, enabled us to perceive and generalize complex patterns in our environment as an evolutionary adaptation [322]. As Agosta and Brooks [322], p. 9 argue in their book, *The Major Metaphors of Evolutionary Transitions*, this generalization and anticipation of complex patterns initially provided early humans with a sense of security, which they identified as ‘good.’ However, the *emergence of complexity* that cannot be generalized frightens us and is perceived as a threat, leading us to label it ‘evil.’ This could mean that our emotions may guide us in recognizing the “boundaries of complexity” regarding our ability to control it or make it less threatening to our existence.

In both cases, everything centres on one key point: the complexity of a system is determined by human nature and human understanding. In other word *ontological holism* boils down to the phenomenological merits of the human mind when confronted with complex “objects,” “entities,” “complex relations,” or whatever they might be. If this assertion is correct, it has significant implications for our understanding of complexity and *ontological holism*, extending well beyond the scope of this paper. Moreover, it could provide new insights into both contemporary and historical debates on the metaphysics of “part–whole” relationships and redefine the search for a definition of life. “Epistemic freedom” in drawing boundaries around biological entities enables biologists to move away from strict functional and morphological depictions of these entities, allowing them to account more flexibly for relations within the living world that often do not respect boundaries. To corroborate this shift from realist ontological holism towards a more pragmatic antirealist one, I must ask: what is science if not a construct of human thought?

Also, when philosophers claim the reality of “many wholes,” as, for example, Julie Zahle [323] and Dave Elder-Vass [324] argue, they face significant challenges in explaining the fragmented worldview of many individual elements of reality. However, to be fair, these authors base their arguments and conclusions in the social sciences, where *individualism* differs from that in the natural world. However, biology is also a specific science that draws its strength from the endless, most beautiful forms and the individual genetic-phenotypic character of each member of a species. The problem is not where we draw boundaries around complex biological (sub)systems. The problem is cognitive, epistemological, and technical in nature, forcing us to accept the reality of different subjective measures of complexity, as elaborated by Gell-Mann [178], or to take Rosenberg’s scepticism [69] and instrumentalism in biology more seriously. In this vein, the question of integration between holistic and emergentist theories in biology must be reframed to include “inside-out” rather than naïve realist “outside-inside” relations.

Even if we compile exhaustive lists of the emergent properties of all the parts that constitute a biological “whole”, we still cannot fully reconstruct a multicellular organism, although there are attempts to create an artificial single cell. Molecules display emergent properties, as demonstrated by Wang et al. [129], and cells, considered as wholes in relation to their organelles and molecules, also have unique emergent properties. However, to achieve more than a mere collection of cells, we must establish every possible relationship and connection (e.g., the relational-processual view of life (see [21])) among these cells simultaneously, with precise timing governed by *closure in production* and *closure of space*. Only then can we account for an “organismic whole” that exhibits emergent properties not present at the cellular and molecular level.

Although we know a great deal about cell biochemistry and molecular biology, we still do not know how to make cells, underscoring the uniqueness of the self-organization of living matter. Indeed, unlike molecules, as Jureček and Švorcová [325], p. 1 state:

“Organic wholes of various levels are defined by informational boundaries and shared evolutionary norms that enable cohesion, cooperation, and distinction from the external environment across diverse biological and cultural systems.”

In plant theoretical biology, authors working on whole-plant physiology since the 70s have increasingly used the term “modules” rather than “parts” to describe the whole-plant organism. Ulrich Lüttge [326] is clear about the meaning of modules:

“Modularity is reductionism and materialism, where modules are considered as building blocks per se.”

This is an old *Newtonian mechanical reductionist* image of plant organization and its physiology. On the other hand, a new picture of “self-organization of modules” in plant physiology and biology in general is gaining ground. By contrast, “self-organization of modules” in living organisms, like plants, generates the emergence of integrated systems with new properties not predicted by the properties of the constituting modules. These lower-level modules are arranged to produce new biological emergent realities or modules. In a more philosophical tone, Ellis [196] termed these models “modular hierarchical structures,” as they provide the basis for complexity, and claims that they account for emergent levels of structure and function based on lower-level networks.

These newly acquired emergent systems become modules for the emergence of new holistic systems at the next higher level [326]. This establishes a hierarchy of networks from molecules, cells, and individuals up to ecosystems, biomes, and the entire biosphere, or Gaia. These higher-level systems above the organism, including the organism itself, are “holobiont-like systems, i.e., central organisms interacting with all their associated organisms as a unit for selection in evolution” [326]. In other words, organisms never evolve in isolation but as holobionts, i.e., as host organisms together with all their associated microorganisms (hologenome concept) [327].

Furthermore, the existence and functioning of biological components depend on the higher-level networks they form and the ongoing management of matter and energy exchange with their environment [328], as stipulated by autopoiesis and holistic–organismic–systems biology. One advantage of the organismic approach, as Bich et al. [328] emphasize, is that it helps us understand how biological systems are integrated into “coherent wholes.” This organismic approach builds on all that has been discussed about self-organization in biology. The only problem with it is that it is much easier to adopt an organismic framework in the case of multicellular organisms, and, as Bich et al. claim, many of these insights are developed through the study of unicellular life.

These challenges concern how cells coexist within more complex entities, where certain features and behaviors are regulated and constrained by the systems they create [327]. Difficulties also arise from the morphogenetic characteristics of multicellular life (morphogenesis), which bring further challenges associated with complex three-dimensional structures (or four-dimensional if time is included) [328]. Changes in cell shape (differentiation) and final position within tissues and organs of plants and animals during development are determined and regulated by genetic regulatory factors, molecular signalling pathways, cell-to-cell communication, and mechanobiology, all influenced by the spatiotemporally ordered expression of genes and the remarkable functional and mechanical properties of the extracellular matrix and the cell cytoskeleton [329,330,331,332]. Moreover, thanks to these processes, a remarkably large number of specialized cell phenotypes form tissues, each with distinct species-specific structural and functional properties, all arising from a single activated oocyte.

### 7.2. The Convergence of Holism and Emergence

*Holism* is not merely a characteristic of emergence; it also establishes a unified connection with it. This important assertion has become central to *holistic systems biology*. Many of the frameworks discussed here have supported this convergence. However, one specific framework that has not yet been mentioned is Bunge’s “systemism” and his CESM model, which provides an alternative link between *reductionism* and *holism*. This model enables self-organization by integrating concepts from quantum physics, physical chemistry, and molecular biology on one hand, and organismic biology and holistic ecology concerning “top-down” constraints and causality on the other. It bridges the artificially separated “bottom-up” and “top-down” approaches in systems biology, addressing the complex challenges researchers face and resolving the conflict between methodologies that emphasize either synthesis or analysis.

Bunge developed the CESM model—comprising *Composition, Environment*, *Structure*, and *Mechanism* [239,253]. Motivated by the limitations of both *holism* and *reductionism* in addressing complexity, Bunge intended his model to play an important role in overcoming both the reductionist neglect of the whole and the holistic view that “only the whole matters, while the parts play a subordinate role” [239].

His “systemic” approach essentially involves analyzing the whole by breaking it down into its parts to study their properties, then reassembling them to understand the system’s behaviour as a whole [239], p. 2. In fact, he depicted the complementary use of holism and reductionism as the only meaningful way to advance complexity science. Both ways of engaging with complex systems are essential for scientific progress; their complementarity is necessary, rather than viewing them as mutually exclusive. As already mentioned, Bunge developed the so-called “ontological systemism”, which builds upon two significant premises and finds its place in the philosophy of medicine [55], p. 3:

(S1) Everything, whether concrete or abstract, is a system or an actual or potential component of a system;
(S2) Systems have systemic (emergent) features that their components lack.

CESM can be described as follows [55], p. 4; [253], pp. 34–35; [239], p. 370:

C(s) = Composition: Collection of all the parts of s (e.g., molecular, cellular, etc.);

E(s) = Environment: Collection of items, other than those of s, that act on or are acted upon by some or all components of s, i.e., immediate surroundings (e.g., family, workplace, etc.);

S(s) = Structure: Collection of relations, in particular bonds, among components of s or between these and items in the environment (e.g., ligaments, hormonal signals, etc.);

M(s) = Mechanism: Collection of processes in s that make it behave as it does, i.e., processes that maintain the system as such (cell division, metabolism, circulation of the blood, etc.).

Based on the CESM model, emergence gives rise to a holistic system. In other words, the CESM model claims to demonstrate the conceptual and real-world isomorphism between emergence and holism, to facilitate the formalization and mathematical depiction of biocomplexity. In light of Elder-Vass and Zahle’s insights into the “many wholes”, for which time emergence and holism converge within a system, the question arises: how many convergences are there in the case of a biological organism and the biosphere? Perhaps there are many temporary convergences which ultimately connect to a single point—in biology, the biosphere, and in philosophy, perhaps the Neoplatonic “One”, above which there are no larger “wholes.” This raises serious philosophical questions, which we do not pursue in this paper.

Furthermore, other models account for the convergence between *holism* and *emergence*. I want to mention all of them and highlight two lesser-known and less popularized: Casper van Elteren [333] and Hans Van Hateren [334]. According to the scientific model provided by Casper van Elteren [333], p. 1, the explanation for their convergence lies in constraints on degrees of freedom that cause system components to interact synergistically and non-trivially, producing novel aggregate outcomes and altering system behaviour. Van Elteren argued that (ibid.):

“Emergence stems not from magical ingredients but from constraints on degrees of freedom, producing outcomes different from—not greater than—the sum of parts.”

For example, a phantom traffic jam or traffic shockwave moving backward due to traffic density can occur even without an accident or bottleneck. Traffic is a dynamic phenomenon in a many-particle system that simulates collective motion (interaction among vehicles when drivers see other vehicles), generating traffic jams similar to phase transitions and pattern formation in non-equilibrium many-particle systems [335], p. 2. Van Elteren’s insights, aligned with the principles of emergence in physics, could lead to a groundbreaking resolution of the longstanding debate between reductionists and holists.

Van Hateren [334] has developed a theoretical system that operates with a high degree of autonomy. It integrates randomness in a targeted and cyclical manner with natural selection, creating a reassuring balance that yields strong emergent properties. Notably, van Hateren’s system, based on standard material components and processes, does not contradict the CCP principle. This theory relates to his [336] framework for understanding the functions of an organism, which can act as independent causal factors. This framework, an estimator theory of mind, aligns with “agential” *goal-directed behavior*, highlighting internal physiological or neural processes that reflect the organism’s fitness and adjust its variability accordingly. The basic conjecture for all life is as follows [337], p. 21:

“All living organisms are proposed here to incorporate an internal process X that makes an estimate x of the organism’s own fitness f, which is produced by an external process F.”

As part of their goal-directedness, this function of estimation is essential for living organisms to account for their own evolutionary fitness, which ultimately governs behaviour and internal physiological processes. Based on this conjecture, Van Hateren derived strong emergent properties and ultimately a holistic theory of consciousness or mind.

What is even more interesting is that Van Hateren’s framework, in his own words, is closely associated with the Darwinian understanding of evolution, which emphasises the differential reproductive success of organisms [337], p. 28. However, he not only established a link between Darwinism and self-organized physiological–thermodynamic attempts to explain the evolution of life, but also facilitated the integration of *agentialism*, *emergence*, and *holism*—a step beyond Bunge. A notable example of his model is the thalamocortical feedback loops, which, from a neurophysiological standpoint, provide a direct link from the periphery (sensory organs) to the cortex, where the auditory, visual, olfactory, and cognitive regions are located. The thalamus is the central sensorimotor relay station, while the cortex processes these stimuli and generates a responsive action if necessary. These structures together are a good example of an estimator of what we perceive as reality, which is then inverted to generate strong emergent consciousness [338].

These convergent models of *holism* and *emergence*, a project started by Bunge and many others, are promising and grounded in realistic assumptions about how various brain subsystems systematically construct consciousness through the coupling of the organism with its environment. Many of these models are currently under development. Emergence and holism will continue to captivate the imagination of scientists and philosophers. As inspiring concepts, they will certainly bring fresh insights to biology, or, conversely, biology may help shed more light on them.

## 8. Conclusions

Understanding the mechanisms underlying the complexity of life is an ever-evolving, perhaps the greatest, frontier in human knowledge, and it has become essential to the pursuit of integrative organismal biology [339]. Along this path, it is crucial to provide conceptual guidance and engage in ongoing reflection on the philosophical and theoretical foundations informed by contemporary complexity science. The science of complexity, together with the study of self-organization, has significantly transformed biology over the past few decades, influencing experimental and theoretical biologists, physicists, chemists, and philosophers in their study of the life sciences. It also offers more scientifically grounded guidance on the longstanding dispute between emergence and holism, making these terms more tangible and rooted in clear theoretical and mathematical-computational foundations. Pattern emergence and models of holistic complex organisms are advancing both clear scientific evidence for an emergentist holistic understanding of life and the much-needed operationalization of these concepts in scientific research practice.

Among many conceptual foundations, the thermodynamic and self-organization paradigm is most intriguing and inspiring, prompting new ideas for explaining the origin, evolution, and development of life. Based on the evidence presented in this paper, self-organization yields a crucial insight: the emergentist-holistic perspective on organisms offers a compelling framework for understanding the origin, evolution, and development of complex biological organisms in all their varieties, shapes, and sizes. Uncovering the roots of self-organization in biology is perhaps the prelude to what I call the science of “self-organizobiology,” a meeting place for different yet complementary ideas based on Prigogine’s “dissipative structures” and all the historical roots or streams of ideas and theories discussed throughout the paper.

This review highlights only a small selection of longstanding and recent ideas for self-organized biological systems at multiple scales, helping biologists, philosophers, and students to navigate the wide range of philosophical ideas that inspire the field. This brief overview of selected conceptual aspects, explicitly or implicitly related to complex biological systems, aims to inspire researchers and practitioners to ground their practices in a sound theoretical perspective and concurrent philosophical ideas. Each conceptual and theoretical framework discussed in the paper is only outlined to provide basic guidelines. For each of the topics mentioned, significant fields of research have involved many authors throughout the turbulent history of 20th-century science. Due to space constraints, they are not mentioned here, but their contributions in these areas are also of the utmost importance for 21st-century biology. At the end of the day, all scientists—biologists, physicists, chemists, medical doctors, philosophers, and others—contribute through various research efforts to the assembly of the mosaic called “life.”

## Data Availability

This paper is conceptual and therefore does not contain any empirical data. All relevant information used in the paper is cited.
